# Host-interactor screens of *Phytophthora infestans* RXLR proteins reveal vesicle trafficking as a major effector-targeted process

**DOI:** 10.1093/plcell/koab069

**Published:** 2021-03-02

**Authors:** Benjamin Petre, Mauricio P Contreras, Tolga O Bozkurt, Martin H Schattat, Jan Sklenar, Sebastian Schornack, Ahmed Abd-El-Haliem, Roger Castells-Graells, Rosa Lozano-Durán, Yasin F Dagdas, Frank L H Menke, Alexandra M E Jones, Jack H Vossen, Silke Robatzek, Sophien Kamoun, Joe Win

**Affiliations:** 1 The Sainsbury Laboratory, University of East Anglia, Norwich Research Park, Norwich, UK; 2 Université de Lorraine, INRAE, IAM, Nancy, France; 3 Department of Life Sciences, Imperial College London, London, UK; 4 Department of Plant Physiology, Institute for Biology, Martin-Luther University Halle-Wittenberg, Halle, Germany; 5 Sainsbury Laboratory, University of Cambridge, Cambridge, UK; 6 Phytopathology Research, Rijk Zwaan Breeding BV, Fijnaart, The Netherlands; 7 Molecular Biology Institute, University of California Los Angeles, Los Angeles, California, USA; 8 Shanghai Center for Plant Stress Biology, CAS Center for Excellence in Molecular Plant Sciences, Chinese Academy of Sciences, Shanghai, China; 9 Gregor Mendel Institute, Austrian Academy of Sciences, Vienna BioCenter, Vienna, Austria; 10 School of Life Sciences, University of Warwick, Coventry, UK; 11 Plant Breeding, Wageningen University and Research, Wageningen, The Netherlands; 12 Ludwig-Maximilian-University of Munich, Munich, Germany

## Abstract

Pathogens modulate plant cell structure and function by secreting effectors into host tissues. Effectors typically function by associating with host molecules and modulating their activities. This study aimed to identify the host processes targeted by the RXLR class of host-translocated effectors of the potato blight pathogen *Phytophthora infestans*. To this end, we performed an in planta protein–protein interaction screen by transiently expressing *P. infestans* RXLR effectors in *Nicotiana benthamiana* leaves followed by coimmunoprecipitation and liquid chromatography-tandem mass spectrometry. This screen generated an effector–host protein interactome matrix of 59 *P. infestans* RXLR effectors x 586 *N. benthamiana* proteins. Classification of the host interactors into putative functional categories revealed over 35 biological processes possibly targeted by *P. infestans*. We further characterized the PexRD12/31 family of RXLR-WY effectors, which associate and colocalize with components of the vesicle trafficking machinery. One member of this family, PexRD31, increased the number of FYVE positive vesicles in *N. benthamiana* cells. FYVE positive vesicles also accumulated in leaf cells near *P. infestans* hyphae, indicating that the pathogen may enhance endosomal trafficking during infection. This interactome dataset will serve as a useful resource for functional studies of *P. infestans* effectors and of effector-targeted host processes.

## Teaser

**One-sentence summary** In planta protein–protein interaction screen of *Phytophthora infestans* RXLR effectors by proteomics approach reveals host vesicle trafficking as a major effector-targeted process.

## Introduction

Plant pathogens reprogram host cells to their advantage to establish successful infections (Dodds and Rathjen, 2010). Understanding how pathogens manipulate their hosts will advance our knowledge of infection processes and help us develop disease-resistant crops (Alfano, 2009; Gawehns et al., 2013; van Schie and Takken, 2014). Pathogens modulate plant cell structure and function by secreting effectors into host tissues. Effectors generally operate by binding or enzymatically modifying host molecules. These host molecules are classified into two major function nal classes: (1) “targets” directly modified by effectors to modulate host processes; (2) “helpers” or “facilitators” coopted by effectors to execute their activities, for instance to traffic within host cells (Win et al., 2012b). Some effectors can be recognized by host immune receptors, consequently triggering an immune response. Such effectors are said to have an avirulence (AVR) activity, as their recognition often results in loss of virulence of the pathogen in hosts carrying a matching immune receptor (Dodds and Rathjen, 2010). Despite major advances in effector biology, our understanding of effector-targeted processes remains fragmentary, and generally centered on suppression of innate immunity (Zheng et al., 2014; Sharpee and Dean, 2016; Toruño et al., 2016; Ren et al., 2019).

Effectors modulate a variety of pathways and therefore can be leveraged as molecular probes to reveal novel and important processes in host cells (Wei et al., 2012; Win et al., 2012b; Lee et al., 2013; Toruño et al., 2016). Pathogen effectors generally associate with host protein complexes to function (Asai and Shirasu, 2015; Boevink et al., 2016). Identifying these host proteins is the first step in unraveling the host processes modulated by effectors. To this end, over the last decade effector biologists have performed large-scale effector–host protein interaction screens using yeast two-hybrid or in planta coimmunoprecipitation/tandem mass spectrometry (coIP/MS) assays (Mukhtar et al., 2011; Weßling et al., 2014; Petre et al., 2015; Petre et al., 2016). While yeast-two hybrid identifies one-to-one protein associations, coIP/MS assays tend to reveal multiple host proteins that associate with a given effector in protein complexes (Win et al., 2011; Petre et al., 2017). This feature makes coIP/MS assays particularly suitable for identifying processes targeted by effectors. Moreover, the increasing availability of plant genome sequences allows for improved proteome predictions that greatly aid IP/MS-based effector interactor searches.

The Irish potato famine pathogen *Phytophthora infestans* (oomycete, Peronosporales) is a major threat to potato (*Solanum tuberosum*) and tomato (*Solanum lycopersicum*) crops worldwide (Fisher et al., 2012; Kamoun et al., 2015; Derevnina et al., 2016). Peronosporales species secrete a superfamily of effector proteins known as RXLR effectors; named after the conserved Arginine-any amino acid-Leucine-Arginine motif that follows the signal peptide of the proteins (Jiang et al., 2008; Win et al., 2012a). The *P. infestans* genome harbors over 500 predicted RXLR effectors that are grouped into ∼150 families and tend to exhibit sequence and expression polymorphisms between pathogen strains (Haas et al., 2009; Cooke et al., 2012; Yoshida et al., 2013; Pais et al., 2018). RXLR effectors are modular proteins with the N-terminal signal peptide and RXLR region mediating secretion and translocation into host cells, and the C-terminal end, often defined by the WY/LWY fold, carrying the effector biochemical activity (Whisson et al., 2007; Win et al., 2012a; He et al., 2019). For example, over a dozen *P. infestans* RXLR effectors have been functionally characterized for their virulence activities (Rovenich et al., 2014; Anderson et al., 2015; Du et al., 2015; Wang et al., 2015; Boevink et al., 2016; Dagdas et al., 2016; Yang et al., 2016; Turnbull et al., 2017; Wang et al., 2018). These effectors bind and alter the stability, activity, or subcellular localization of a diversity of host proteins, including proteases, kinases, phosphatases, transcription factors, ubiquitin ligases, RNA binding proteins, autophagy-related proteins, and vesicular trafficking-associated proteins. The emerging picture is that *P. infestans* RXLR effectors modulate multiple host processes to the pathogen’s benefit. Nonetheless, systematic interactome screens between *P. infestans* RXLR effectors and host proteins have not been reported to date.

Many plant pathogenic and symbiotic microbes produce specialized hyphae that invade host cells but remain enveloped by newly synthesized host-derived membranes (Oliveira-Garcia and Valent, 2015; Ivanov et al., 2019; Bozkurt and Kamoun, 2020). Processes taking place at this host–pathogen interface are thought to have a major impact on the outcome of the interaction. The mechanisms underlying the biogenesis and functions of host–microbe interfaces remain poorly understood although recent advances have been made (Bozkurt and Kamoun, 2020; Rausche et al., 2020). Well-studied examples of these specialized hyphae are haustoria formed by *P. infestans* and other filamentous pathogens. During infection, haustoria enter the plant cell cavity and become surrounded by the plant-derived extrahaustorial membrane (EHM). *Phytophthora infestans* effectors secreted at this pathogen–host interface are thought to reprogram multiple processes in the invaded (haustoriated) host cells (Bozkurt et al., 2012; Dagdas et al., 2016; Dagdas et al., 2018). In addition, a number of *P. infestans* RXLR effectors, for example, AVRblb2, AVR1, AVR2, and PexRD54, focally accumulate around haustoria when they are heterologously expressed in host cells (Bozkurt et al., 2011; Saunders et al., 2012; Dagdas et al., 2018; Wang et al., 2018). These findings, along with related cell biology and biochemical studies, indicate that *P. infestans* massively reprograms host membrane trafficking during infection (Du et al., 2015; Tomczynska et al., 2018). Membrane trafficking perturbations of haustoriated cells include the rerouting to the haustorial interface of components of the endocytic pathways (Lu et al., 2012; Bozkurt et al., 2015), autophagy machinery (Dagdas et al., 2018; Pandey et al., 2020), and chloroplasts (Toufexi et al., 2019).

This study aims to identify the host plant proteins that are associated with a representative set of *P. infestans* RXLR effectors to generate a pathogen–host protein interactome and gain an overview of the diversity of effector-targeted processes in this pathosystem. To achieve our aim, we performed an in planta coIP/MS screen in the model plant *Nicotiana benthamiana*, which has emerged as an optimal experimental system for cell biology and biochemistry studies of *P. infestans*–host interactions (Bozkurt and Kamoun, 2020). In addition, *N. benthamiana* is particularly appropriate for large-scale coIP/MS screens thanks to rapid transient protein expression using *Agrobacterium tumefaciens* (agroinfiltration) and the availability of a genome sequence that improves MS proteome predictions (Goodin et al., 2008; Derevnina et al., 2019; Zess et al., 2019). Using this approach, we generated a host protein–pathogen effector interactome matrix of 59 *P. infestans* RXLR effectors x 586 *N. benthamiana* proteins. This interactome revealed 35 candidate effector-targeted processes in *N. benthamiana*. We further characterized the PexRD12/31 family of RXLR-WY effectors, which associate and colocalize with components of the vesicle trafficking machinery. One member of this family, PexRD31, increased the number of 2xFYVE-GFP labeled vesicles in *N. benthamiana* cells. We also noted that *P. infestans* alters the number and the distribution of 2xFYVE-GFP vesicles in *N. benthamiana* leaves, indicating that the pathogen dramatically alters host endosomal trafficking during infection.

## Results

### In planta coIP/MS assays reveal host proteins associated with *P. infestans* RXLR effectors

To identify host protein complexes associated with representative *P. infestans* RXLR effectors, we performed a protein–protein interaction screen in planta using a pipeline similar to the one we previously described for candidate effector proteins of the poplar leaf rust fungus (Petre et al., 2015). We first selected 66 effectors from 14 prominent RXLR families, which are enriched in effectors that are (1) transcriptionally induced during plant infection and (2) carrying AVR activities (Table 1; Oh et al., 2009; Vleeshouwers et al., 2011). We then expressed N-terminal FLAG -tagged effector proteins individually in *N. benthamiana* leaf cells by agroinfiltration and performed anti-FLAG coIP/MS to identify host protein complexes associated with each effector (Supplemental Figure S1, see “Methods” for details). We evaluated the expression of the effector fusions by analyzing immunoprecipitated protein mixtures using sodium dodecyl sulfate-polyacrylamide gel electrophoresis (SDS-PAGE) coupled with colloidal Coomassie blue (CCB) staining (Supplemental Figure S2). Out of 66 effectors, 59 accumulated to adequate levels for coIP and identification by MS. Spectral searches against the *N. benthamiana* proteome revealed 965 host protein interactors for the 59 effectors (Supplemental Data Set S1A).

**Table 1:** *Phytophthora infestans* RXLR effectors used in coIP study

Effector	GenBank Accession	Family^a^	Size (full length)	Insert peptide	WY fold^b^	Cell death^c^	MS Data^d^	Whole lane (or) bands^e^	Reference
AVR2	QBB68791	AVR2	116	66-116	0	No	No	N.D.	Gilroy et al. (2011)
PexRD11	ACX46536	AVR2	114	22-114	0	No	Yes	Whole lane	Oh et al. (2009)
PexRD11sh	ACX46536	AVR2	114	64-114	0	No	Yes	Whole lane	Oh et al. (2009)
PITG_05121	XP_002998822	AVR2	114	67-114	0	No	No	N.D.	Haas et al. (2009)
PITG_06077	XP_002998270	AVR2	118	67-118	0	No	No	N.D.	Haas et al. (2009)
PITG_07500	XP_002904502	AVR2	118	66-118	0	Yes	Yes	Whole lane	Haas et al. (2009)
PITG_08278	XP_002903684	AVR2	118	66-118	0	No	Yes	Whole lane	Haas et al. (2009)
PITG_13936	XP_002899599	AVR2	96	64-96	0	No	Yes	Whole lane	Haas et al. (2009)
PITG_13940	XP_002899603	AVR2	114	64-114	0	No	Yes	Whole lane	Haas et al. (2009)
PITG_15972	XP_002898185	AVR2	94	66-94	0	No	Yes	Whole lane	Haas et al. (2009)
PITG_19617	XP_002996938	AVR2	118	66-118	0	No	No	N.D.	Haas et al. (2009)
PITG_21949	XP_002996876	AVR2	114	64-114	0	No	Yes	Whole lane	Haas et al. (2009)
PITG_22975	XP_002899618	AVR2	114	64-114	0	No	Yes	Whole lane	Haas et al. (2009)
AVR3a	E2DWQ7	AVR3a	147	22-147	1	No	Yes	Whole lane	Amstrong et al. (2005)
Pex147-2	XP_002898841	AVR3a	148	22-148	1	No	No	N.D.	Amstrong et al. (2005)
Pex147-3	XP_002898843	AVR3a	147	22-147	1	No	Yes	Bands	Amstrong et al. (2005)
AVR4	XP_002904419	AVR4	287	22-287	4	No	Yes	Bands	van Poppel et al. (2008)
AVRblb1	XP_002895051	AVRblb1	152	22-152	0	Yes	Yes	Whole lane	Vleeshouwers et al (2008)
PexRD39	ACX46588	AVRblb2	92	15-92	0	No	No	N.D.	Oh et al. (2009)
PexRD39sh	ACX46588	AVRblb2	92	39-92	0	No	Yes	Whole lane	Oh et al. (2009)
PexRD40	ACX46596	AVRblb2	92	15-92	0	No	Yes	Whole lane	Oh et al. (2009)
PexRD40_34aa	ACX46596	AVRblb2	92	59-92	0	No	No	N.D.	Oh et al. (2009)
PexRD40sh	ACX46596	AVRblb2	92	39-92	0	No	No	N.D.	Oh et al. (2009)
PexRD41	ACX46573	AVRblb2	84	22-105	0	No	Yes	Bands	Oh et al. (2009)
PexRD45	ACX46578	AVRblb2	90	19-106	0	No	No	N.D.	Oh et al. (2009)
PexRD46	ACX46579	AVRblb2	84	22-105	0	No	No	N.D.	Oh et al. (2009)
PITG_04086	XP_002905796	AVRblb2	100	47-100	0	No	Yes	Whole lane	Haas et al. (2009)
PITG_04097	XP_002905808	AVRblb2	107	44-107	0	No	Yes	Whole lane	Haas et al. (2009)
PITG_04194	XP_002905878	AVRblb2	107	44-107	0	No	Yes	Whole lane	Haas et al. (2009)
PITG_18675	XP_002997305	AVRblb2	107	44-107	0	No	Yes	Whole lane	Haas et al. (2009)
PITG_18683	XP_002997312	AVRblb2	100	47-100	0	No	Yes	Whole lane	Haas et al. (2009)
PITG_20300	XP_002895918	AVRblb2	100	47-100	0	No	No	N.D.	Haas et al. (2009)
PITG_20301	XP_002895919	AVRblb2	100	47-100	0	No	No	N.D.	Haas et al. (2009)
PITG_20303	XP_002895919	AVRblb2	100	47-100	0	No	Yes	Whole lane	Haas et al. (2009)
PITG_07689	XP_002904634	AVRpur	104	27-104	0	No	Yes	Whole lane	Haas et al. (2009)
PITG_07689	XP_002904634	AVRpur	104	27-104	0	No	Yes	Whole lane	Haas et al. (2009)
PITG_16275	XP_002897665	AVRpur	170	25-170	0	No	Yes	Whole lane	Haas et al. (2009)
PITG_16294	XP_002897362	AVRvnt1	153	24-153	0	No	No	N.D.	Haas et al. (2009)
PITG_18880	XP_002997157	AVRvnt1	154	23-154	0	No	Yes	Whole lane	Haas et al. (2009)
PITG_18880-4	XP_002997157	AVRvnt1	154	23-154	0	No	Yes	Whole lane	Haas et al. (2009)
NUK10	XP_002898730	PexRD1	213	20-213	2	No	No	Single band	Haas et al. (2009)
NUK10-NLS	XP_002898730	PexRD1	213	20-213	2	No	No	Single band	Haas et al. (2009)
PexRD2	XP_002894954	PexRD2	121	21-121	1	Yes	Yes	Whole lane	Oh et al. (2009)
PITG_14787	XP_002898994	PexRD2	130	57-130	2	No	No	N.D.	Haas et al. (2009)
PexRD3	ACX46525	PexRD3	168	25-168	2	No	Yes	Whole lane	Oh et al. (2009)
PexRD6	AAA21423	PexRD6	152	22-152	1	Yes	No	N.D.	Oh et al. (2009)
PexRD8	XP_002898614	PexRD8	142	23-142	0	No	No	N.D.	Oh et al. (2009)
PexRD12 (PITG_16245)	XP_002897644	PexRD12/31	115	62-115	1	Yes^f^	No	N.D.	Haas et al. (2009)
PexRD31 (PITG_23074)	XP_002897462	PexRD12/31	119	66-119	1	Yes	Yes	Whole lane	Oh et al. (2009)
PITG_16233	XP_002897640	PexRD12/31	123	71-123	1	No	Yes	Whole lane	Haas et al. (2009)
PITG_16235	XP_002897637	PexRD12/31	129	77-129	1	No	Yes	Whole lane	Haas et al. (2009)
PITG_16242	XP_002897642	PexRD12/31	129	23-129	1	No	Yes	Whole lane	Haas et al. (2009)
PITG_16243	XP_002897643	PexRD12/31	119	66-119	1	Yes	Yes	Whole lane	Haas et al. (2009)
PITG_16248	XP_002897647	PexRD12/31	119	66-119	1	Yes	No	N.D.	Haas et al. (2009)
PITG_16409	XP_002897637	PexRD12/31	129	77-129	1	No	Yes	Whole lane	Haas et al. (2009)
PITG_16428	XP_002897468	PexRD12/31	142	75-142	1	Yes	Yes	Whole lane	Haas et al. (2009)
PITG_20336	XP_002897640	PexRD12/31	123	71-123	0	No	Yes	Whole lane	Haas et al. (2009)
PITG_23069	XP_002897638	PexRD12/31	119	66-119	1	Yes	Yes	Whole lane	Haas et al. (2009)
PITG_05911	XP_002998153	PexRD18	135	67-135	1	No	Yes	Whole lane	Haas et al. (2009)
PITG_05912	XP_002998154	PexRD18	135	67-135	1	No	Yes	Whole lane	Haas et al. (2009)
PITG_05918	XP_002998156	PexRD18	135	67-135	1	No	Yes	Whole lane	Haas et al. (2009)
PITG_22089	XP_002894699	PexRD18	135	67-135	1	No	Yes	Whole lane	Haas et al. (2009)
35a12_90128	N/A	PexRD35a12	143	67-143	1	No	Yes	Whole lane	This study
35a12_Cu10	N/A	PexRD35a12	143	67-143	1	No	Yes	Whole lane	This study
PexRD36	ACX46558	PexRD36	106	23-106	1	Yes	Yes	Bands	Oh et al. (2009)
PexRD54	XP_002903599	PexRD54	381	76-381	5	Yes	Yes	Whole lane	Oh et al. (2009)

^a^ Family clustering was based on Haas et al. (2009).

^b^ Presence of WY domain was predicted using HMMER version 3.2.1 software and the WY-fold HMM reported in Boutemy et al. (2011).

^c^ Cell death observed 3 days post infiltration using pTRBO vectors.

^d^ MS data successfully collected.

^e^ Whole lane, whole lane of protein gel was cut into gel slices and all slices were submitted for MS; Bands, each visible Coomassie-stained protein bands on the gel for each effector were cut and submitted for MS; N.D., No band was present, and no data could be collected.

^f^ Cell death was observed only when N-terminally tagged with GFP and mCherry fluorescent proteins.

**Figure 1:**
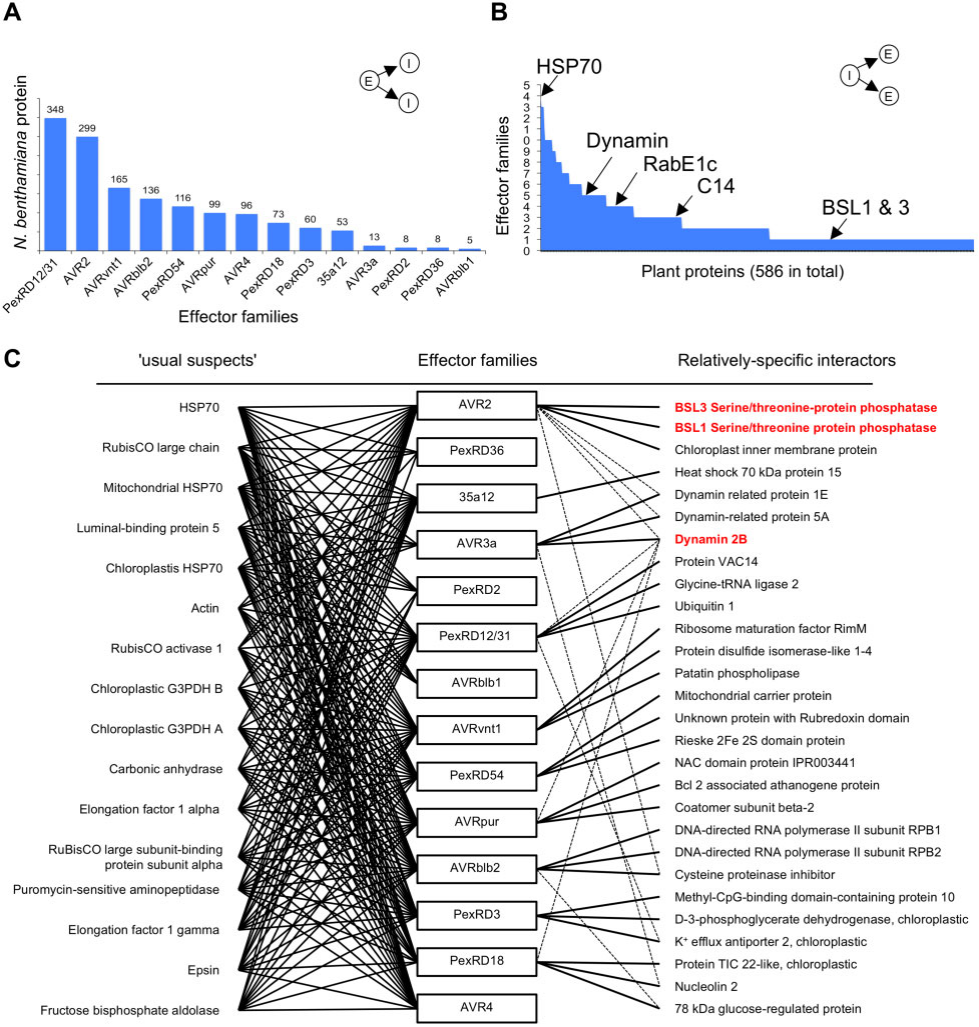
Overview of the *P. infestans* RXLR effector × *N. benthamiana* protein interactome. A, Number of plant proteins that associate with each of the tested 14 RXLR effector families of *P. infestans*. E = effector family; I = host interactor. B, Number of effector families that associate with each *N. benthamiana* protein. For example, HSP70 was associated with all 14 effector families while previously experimentally validated interactors BSL1, BSL3, C14, dynamin, and RabE1c associated with one to five effector families. C, Interactome network of most promiscuous *N. benthamiana* proteins (also referred to as the “usual suspects,” shown on the left) and a selection of relatively-specific interactors (on the right) for each effector family. The usual suspects include plant proteins associated with more than nine different effector families. The relatively-specific set includes top three *N. benthamiana* proteins identified in coIP/MS from each effector family by following criteria: have at least three peptides hits; have the maximum peptide hits in coIP/MS of the effector family they associate within the dataset; and show association with the least number (up to five) of effector family. Effector families PexRD36, PexRD2, AVRblb1, and AVR4 did not show association with plant proteins that fulfill the aforementioned criteria. The dotted lines indicate associations that do not have the maximum number of peptide hits in coIP/MS within the dataset. The plant proteins shown in red bold text have been experimentally validated for their association with respective effector families.

Next, we deconvoluted the 59 × 965 matrix to facilitate the interpretation of the results. First, given that multiple RXLR effectors are related and analyses of individual effectors would skew the results toward the largest families, we merged the data for the 59 individual effectors into 14 sequence-related families (Supplemental Data Set S2A). In total, 7 effectors remained as singletons whereas the others grouped into families that range in size from 2 to 16 (AVRblb2 being the largest family; Table 1). Second, to further reduce the matrix complexity, we (1) filtered the proteins with the criteria of having at least two peptide hits that have equal or >90% probability and an ion score of ≥40 in their MS spectral matches, and (2) clustered host protein into groups having at least 80% identity over at least 80% of length. This yielded a total of 586 host proteins, which were represented by a single protein entry to make a consolidated interactome matrix (Supplemental Data Set S2A). We used this interactome network for further analyses. On average, a given effector family associated with 105 host proteins (range 5–348), with the PexRD12/31 family associating with the largest number of proteins (Figure 1A). *Nicotiana benthamiana* interactor proteins associated on average with 2.5 effector families (Figure 1B).

### *Phytophthora infestans* RXLR effector families associate with host protein complexes that include previously validated interactors

One limitation of affinity purification-MS assays is that they tend to yield false positives, notably abundant and sticky proteins (Mellacheruvu et al., 2013; Petre et al., 2015). Given that the same false positives tend to appear in independent experiments, interactors that are specific to a smaller subset of bait proteins are more likely to be biologically relevant. We, therefore, flagged the most promiscuous interactors that associated with >9 effector families as likely representing false positives (Figure 1C). These 16 “usual suspect” interactors overlap with the list previously reported by Petre et al. (2015) for *N. benthamiana* interactors of candidate effectors from the poplar rust fungus.

Five host interactors of *P. infestans* RXLR effectors in our interactome matrix have been previously validated (Supplemental Table S1). The cysteine protease RD21a (C14) associates with members of the AVRblb2 family (Bozkurt et al., 2011; Wang et al., 2015), the BRASSINOSTEROID-INSENSITIVE1-SUPPRESSOR1-like serine/threonine protein phosphatases BSL1 and BSL3 associate with members of the AVR2 family (Saunders et al., 2012), dynamin 2B associates with the AVR3a family (Chaparro-Garcia et al., 2015) and Ras-related protein RabE1c (also known as Rab8a) associates with PexRD54 (Pandey et al., 2020; Supplemental Table S1; Figure 1C). These validated host interactors associated with anywhere from 1 to 5 effector families in our experiments (Supplemental Table S1). Therefore, we reasoned that host interactors showing association with five or fewer effector families would likely represent relatively specific interactors. Using this cut-off, we identified a set of 497 near-specific host interactors in our matrix (Supplemental Data Set S2A). We conclude that the interactome matrix we generated is likely to contain biologically relevant host protein–effector associations and warrants further investigation.

### Interactome network analyses reveal putative host processes targeted by *P. infestans* RXLR effector

**Figure 2:**
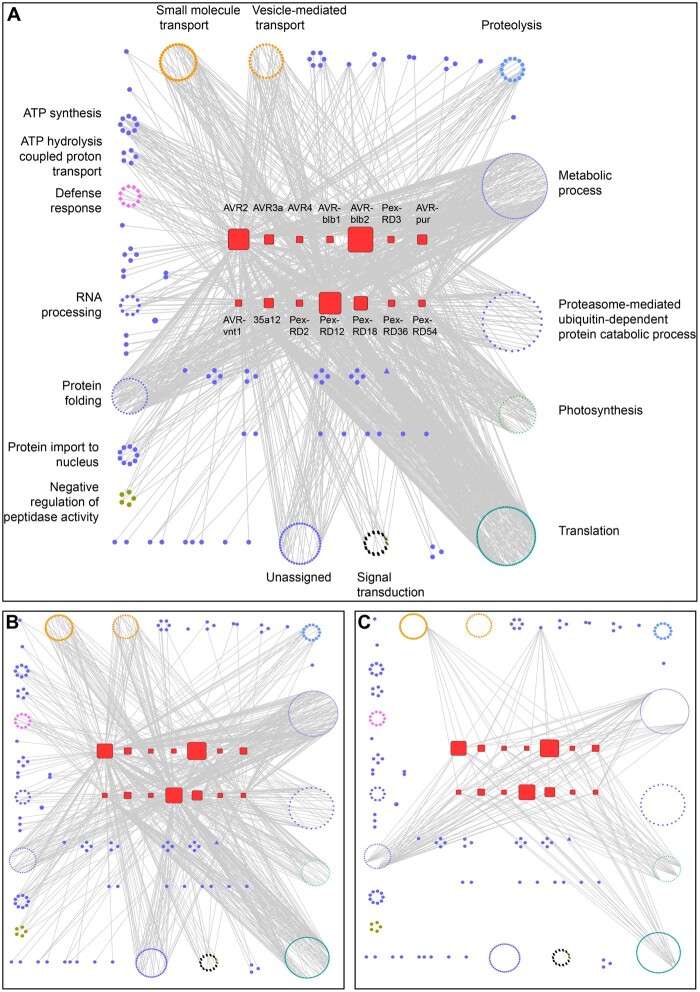
Interactome network analyses reveal host biological processes likely targeted by *P. infestans* RXLR effectors. A, General network overview of the association between the RXLR effector families and the host interactors grouped according to their predicted biological processes using GO terms. B, Same as (A), with only the relatively specific associations (i.e. host interactors associated with <5 effector families) depicted. C, Same as (A), with only the redundant associations (i.e. host interactors associated with >9 effector families) depicted.

The host interactors of the RXLR effectors can serve as proxies to identify candidate host processes targeted by *P. infestans*. To this end, we categorized the 586 host interactors according to their Gene Ontology (GO) terms (Supplemental Data Set S2B) and built a GO-based interactome network from protein-to-protein binary interactions (Figure 2A;Supplemental Table S2). Nodes represent plant proteins or effector families and edges represent the interaction between two proteins. Classification of the host interactors into putative functional GO categories revealed about 35 biological processes that are potentially targeted by effectors (Supplemental Table S2). The network revealed that 50% of the interactions involved host proteins from three main GO categories: translation, metabolic process, and photosynthesis. Some of these may be false positives. For example, we anticipated that proteins involved in translation would associate with effectors during in planta expression and may be difficult to study as bona fide effector targets.

To clarify the network topology, we generated subnetworks by mapping the 497 near-specific host interactors that associated with less than five effector families (Figure 2B). This subnetwork revealed that edges from host proteins involved in some of the plant processes converged on a particular effector family. For example, the edges from plant proteins involved in “small molecule transport” and “vesicle-mediated transport” processes converged on the PexRD12/31 family of effectors; the edges from proteins assigned to the “protein import to nucleus” process converged on the AVRvnt1 family; and the edges from proteins in “signal transduction” and “proteolysis” processes converged on the AVR2 family (Figure 2B;Supplemental Data Set S3). To visualize these connections, we generated subnetworks for selected processes (Supplemental Figure S3; Supplemental Data Set S3). These subnetworks revealed more detailed associations between individual host proteins involved in the biological process and effector families. For example, 24 out of the 32 members of the “vesicle-mediated transport” process associated with the PexRD12/31 family, and seven out of nine members of the “protein import to nucleus” associated with the AVRvnt1 family. The observation that multiple proteins assigned to the same host biological process associated predominantly with single effector families suggests that these host processes constitute targets of these effectors.

We also generated an interaction subnetwork for the 16 most promiscuous interactors that showed association with more than nine effector families (Figure 2C). This subnetwork revealed that the promiscuous host interactors are distributed across different biological processes with edges radiating toward different effector families in an indiscriminate manner, suggesting that these interactions are non-specific. Therefore, we conclude that the GO-based interactome network analysis was useful in flagging host processes likely targeted by *P. infestans* RXLR effectors as well as identifying potential non-specific interactions.

### PexRD12/31 proteins form a complex family of *P. infestans* RXLR-WY effectors

Given the prominence of vesicle-mediated trafficking as a target of the PexRD12/31 family and considering that the functions of this large effector family remain largely unknown, we decided to further characterize this family. We reasoned that PexRD12/31 effectors could serve as useful probes to shed further light on how *P. infestans* manipulate host vesicular trafficking. The PexRD12/31 family comprises 19 predicted members in the genome of *P. infestans* strain T30-4 (Haas et al., 2009). Two family members, PexRD12 (PITG_16245) and PexRD31 (PITG_23074), were previously reported in *P. infestans* isolate 88069 and included in in planta screens for AVR activities (Vleeshouwers et al., 2008; Oh et al., 2009). PexRD12 is recognized by Rpi-chc1, which is encoded by a *Solanum chacoense* resistance gene against *P. infestans* (Vossen et al., 2011).

**Figure 3:**
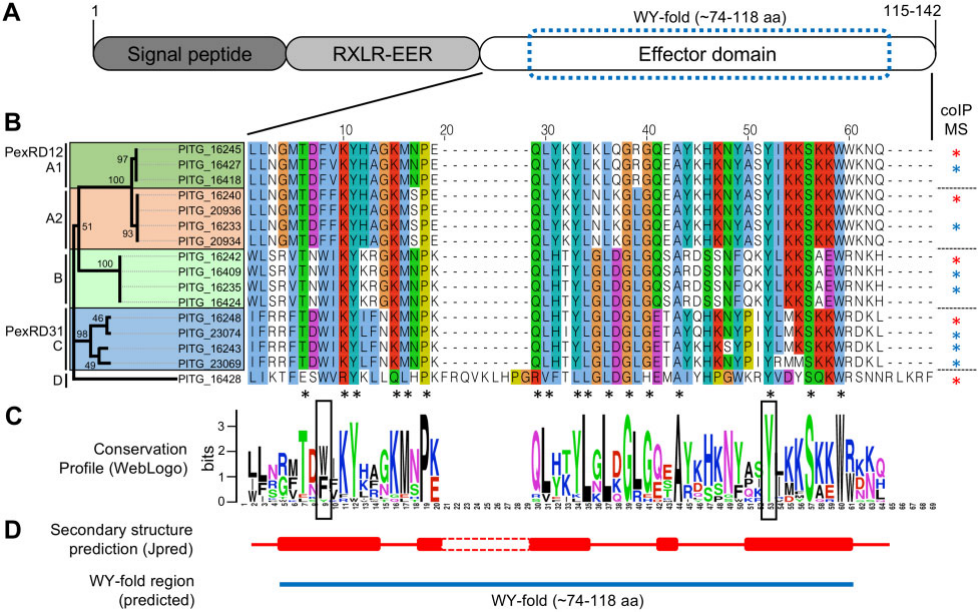
*Phytophthora infestans* PexRD12/31 family of RXLR effectors group into four distinct classes. A, Schematic diagram of the canonical protein domain organization of PexRD12/31 proteins. Numbers indicate minimal and maximal peptide length of the effectors. B, Phylogenetic tree and amino acid alignment of the C-terminal domain of the 16 members of the PexRD12/31 family. The family consists of four distinct groups (A1, A2, B, and C) and PITG_16428 on its own in D. The phylogenetic relationship within the PexRD12/31 family members was inferred using the Neighbor-Joining method. The unrooted optimal tree is shown, but not to scale. The bootstrap values (100 replicates) are shown next to the nodes. This analysis involved 16 amino acid sequences. All positions containing gaps and missing data were eliminated. There was a total of 54 positions in the final dataset. Evolutionary analyses were conducted in MEGA X. Amino acid residues are colored according to the ClustalX scheme. The black asterisks indicate the amino acid residues that are conserved in ≥90% of the sequences. The blue asterisks indicate effectors used for the first coIP/MS screen; the red asterisks indicate effectors used for both the coIP/MS screen and follow-up experiments. C, WebLogo conservation profile of the sequence alignment shown in (B). D, Jpred4-predicted alpha-helices and predicted WY-fold in PexRD12/31 family members. Solid red cylinders indicate predicted helices based on the sequences of PITG_16424 and PITG_23074. The predicted WY-fold region is indicated by a blue bar.

Sequence analyses of the 19 predicted genes revealed that two members (PITG_23230 and PITG_20336) lack the effector domain and one divergent member (PITG_09577) lacks the signature RXLR-EER motif. To evaluate the sequence diversity of the remaining 16 family members, we compared the amino acid sequence of their effector domains (Figure 3). Phylogenetic analyses grouped 15 of the 16 members into four clades supported by bootstrap values >90%, while one divergent member (PITG_16428) contained a 10 amino acid insertion in the effector domain and did not cluster with any of the other proteins (Figure 3; Supplemental File S1). The effector domains of the canonical family members comprise 62 amino acids and all 16 proteins contained a single predicted WY-fold based on HMMER searches (E value < 1.8^−07^; Boutemy et al., 2011). Proteins within each of the four clades displayed an average pairwise amino acid identity of >95% in their effector domains. We conclude that the PexRD12/31 family of *P. infestans* RXLR-WY effectors is structured into four clades of highly similar proteins with PITG_16428 as a fifth divergent member of the family.

### PexRD12/31 effectors consistently associate with vesicle trafficking-related host proteins in independent coIP/MS experiments

To further explore the association between PexRD12/31 effectors and host proteins implicated in vesicle trafficking, we fused the effectors to green fluorescent protein (GFP) and used them in additional coIP/MS experiments. We selected one representative from each of the four PexRD12/31 clades: PexRD12, PexRD31, PITG_16242, and PITG_16428 (Figure 3). We expressed the GFP effector fusion proteins in *N. benthamiana* leaf cells by agroinfiltration and performed coIP/MS using anti-GFP antibodies under the stringent conditions described in the methods (Supplemental Data Set S4). All the fusion proteins could be detected after coIP by either SDS-PAGE CCB staining, MS, or both, indicating effective expression and IP (Supplemental Figure S4A). These assays yielded 24 out of the 30 vesicle trafficking-related host proteins that we previously identified in the anti-FLAG coIP/MS screen (Supplemental Table S3). Among these 24 proteins, 16 associated with no more than four bait proteins throughout our laboratory dataset of over a hundred independent coIP/MS assays (Supplemental Data Sets S1A and S2A; Petre et al., 2015; Petre et al., 2016). We conclude that these 16 *N. benthamiana* proteins are relatively specific interactors of PexRD12/31 effectors in coIP/MS assays.

### PexRD12/31 effectors associate with *N. benthamiana* R-SNARE protein of the VAMP72 family

Vesicle-associated membrane proteins (VAMPs; also known as R-SNAREs) are components of secretory vesicles and endosomes that mediate vesicle fusion. VAMPs have been implicated in plant-pathogen response (Collins et al., 2003; Kwon et al., 2008; Yun and Kwon, 2017) but are not known to be targeted or associated with pathogen effectors. Our FLAG coIP/MS assays identified a protein annotated as VAMP 7B (Sol Genomics Network (SGN) accession NbS00022342g0004) by two peptide hits in Mascot searches from IP of effector PITG_16428, a member of PexRD12/31 family (Supplemental Figure S5). In addition, other PexRD12/31 effectors were also shown to consistently and specifically associate with VAMP 7B in the GFP coIP/MS assays (Supplemental Table S3).

VAMPs occur as multiple paralogs in *N. benthamiana* (Supplemental Figure S5). To further characterize the sequence of NbS00022342g0004 we performed BLASTP (BLAST 2.9.0+; Altschul et al., 1997) searches against the *Arabidopsis thaliana* proteome version Araport11 in which VAMPs are comprehensively annotated (The Arabidopsis Information Resource, https://www.arabidopsis.org). The top hit was AT1G04760, annotated as AtVAMP726, with an E value of 2^−126^ and a score of 357. To determine the relationship between NbS00022342g0004 and *A. thaliana* VAMPs, we extracted all members of AtVAMP72 family (AtVAMP721-727) from Araport11 and 14 VAMP72-like amino acid sequences from the proteome of *N. benthamiana* (SGN, version 0.44). Phylogenetic analyses of these sequences revealed that NbS00022342g0004 does not have a clear ortholog in *A. thaliana* within the VAMP72-family (Supplemental Figure S5; Supplemental File S2) and therefore we decided to refer to it from here on as NbVAMP72x.

**Figure 4:**
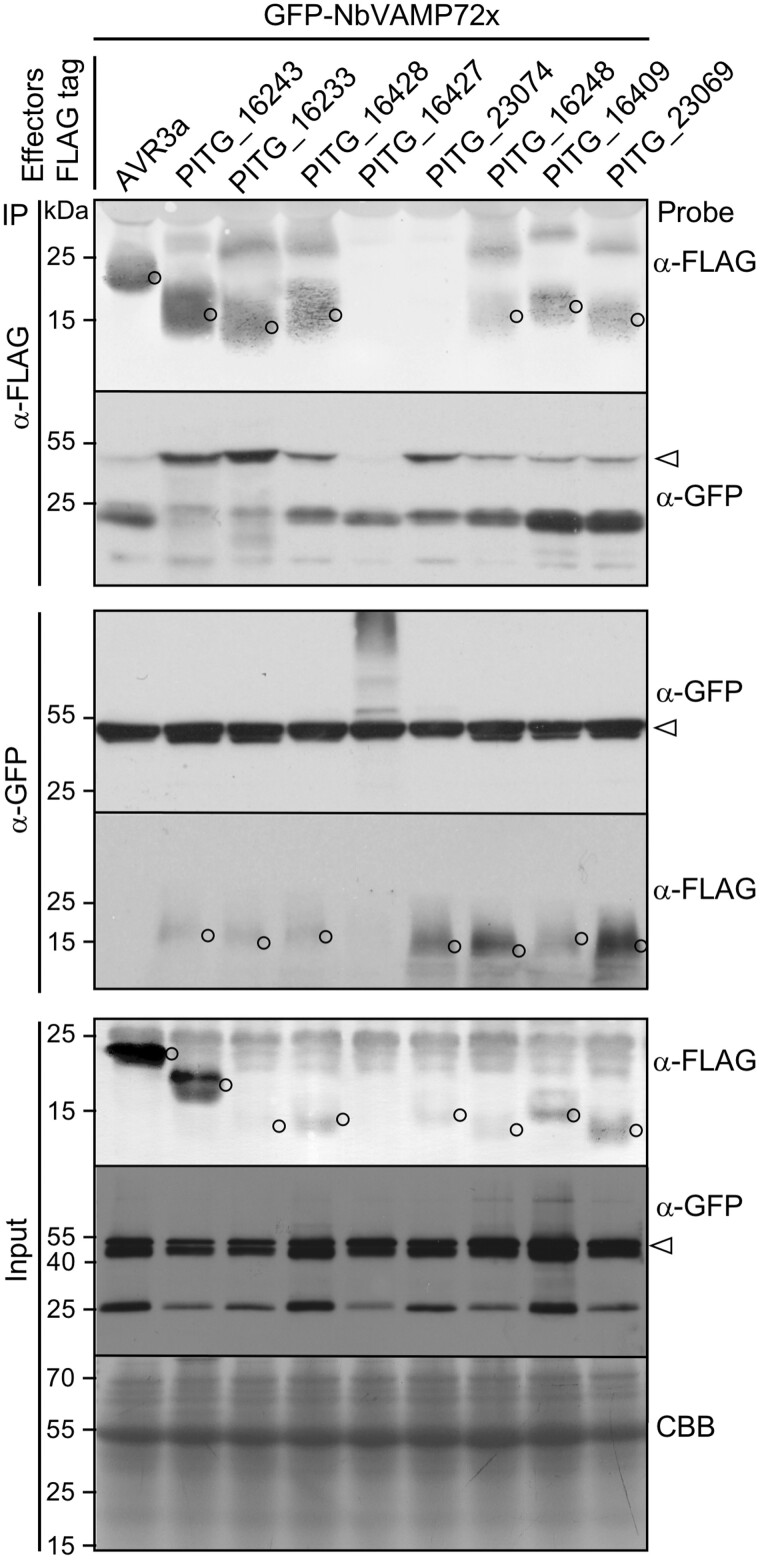
PexRD12/31 effectors associate with NbVAMP72x in planta. Immunoblots show FLAG-tagged PexRD12/31 family effector fusion proteins coimmunoprecipitate with GFP-tagged NbVAMP72x transiently coexpressed in *N. benthamiana.* Approximate molecular weights of the proteins are shown on the left. Open arrowhead shows the expected size of the NbVAMP72x bands and open circles show the expected sizes of the effector bands.

To further evaluate whether PexRD12/31 effectors physically associate with NbVAMP72x in planta, we combined coIP and immunoblot assays. We coexpressed eight FLAG-tagged PexRD12/31 effectors with GFP-NbVAMP72x in *N. benthamiana* leaf cells and performed IPs with anti-FLAG and anti-GFP antibodies bound to agarose beads. These coIP assays revealed that seven tested PexRD12/31 effectors associated with NbVAMP72x in planta (Figure 4; Supplemental Figure S5C). One effector, PITG_16427, did not associate with NbVAMP72x. However, PITG_16427 did not accumulate to detectable levels in the input samples indicating that the protein is not stable in planta and making the IP assay inconclusive (Figure 4). The negative control FLAG-AVR3a, which did not associate with NbVAMP72x in the coIP/MS screens described earlier, also did not associate with NbVAMP72x in the coIP/immunoblot experiments. We conclude that members of the PexRD12/31 family associate with NbVAMP72x in planta.

### PexRD12/31 effectors mainly accumulate at the host plasma membrane

To determine the subcellular localization of PexRD12/31 effectors in plant cells, we expressed the GFP N-terminally tagged PexRD12, PexRD31, PITG16242, and PITG16428 described above in *N. benthamiana* leaf cells by agroinfiltration and performed live cell imaging by confocal microscopy. GFP-tagged PexRD31, PITG16242, and PITG16428 produced informative fluorescent signals that distributed mainly at the cell periphery (Supplemental Figure S6A–C). In addition, the GFP-PexRD31 fluorescent signal accumulated sharply around the nucleus and in small and mobile puncta, which were well visible at high magnification and while imaging large areas of cytosol (at cell top for instance). GFP-PexRD12 was not informative as it produced a weak fluorescent signal and triggered a necrotic response in leaves that interfered with the imaging.

To evaluate the robustness of these observations, we performed similar live cell imaging experiments with effectors N-terminally tagged with the fluorescent protein mCherry (Supplemental Figure S6D–F). We observed similar patterns for PexRD31, PITG16242, and PITG16428 with the fluorescence signal accumulating mainly at the cell periphery. Here too, mCherry-PexRD12 triggered a necrotic response with no clear fluorescent signal. Interestingly, as observed with the GFP fusion, mCherry-PexRD31 labeled the nuclear periphery and mobile cytosolic puncta in addition to the cell periphery (Figure 6A). We also documented these mobile signals in a movie (Supplemental Movie S1).

**Figure 5:**
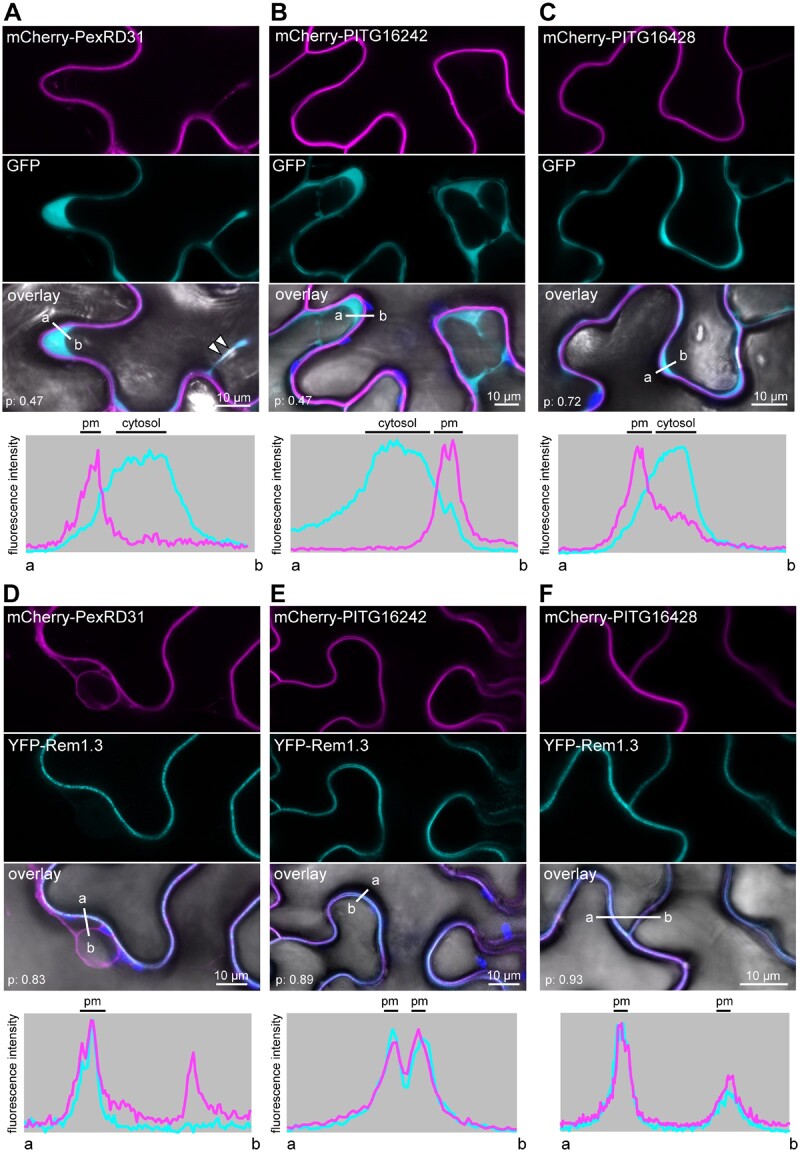
PexRD12/31 effectors accumulate at the host pm. mCherry-PexRD31, mCherry-PITG16242, and mCherry-PITG16428 were coexpressed with free GFP or with the pm marker YFP-Rem1.3 via agroinfiltration in *N. benthamiana* leaves. Live-cell imaging was performed with a laser-scanning confocal microscope 3 days after infiltration. A–F, Confocal microscopy images of *N. benthamiana* leaf epidermal cells coexpressing mCherry-PexRD31, mCherry-PITG16242, or mCherry-PITG16428 with free GFP (A–C), or pm marker YFP-Rem1.3 (D–F). All three effectors show PM localization, while mCherry-PexRD31 also accumulated in cytosolic puncta (white arrowheads). Images are single optical sections of 0.8 μm. The overlay panel combines GFP (A–C) or YFP (D–F), mCherry, chlorophyll, and bright-field images. The lower panels show relative fluorescence intensity plots of the GFP (A–C) or YFP (D–F) (cyan) and the mCherry (magenta) signals along the line from a to b depicted in the corresponding overlay panels. Colocalization scores (p: Pearson’s *R*-value) are indicated in overlay images.

To determine whether PexRD31, PITG16242, and PITG16428 accumulate at the plasma membrane (pm), we coexpressed the mCherry fusion proteins with the pm marker protein YFP-Rem1.3 in *N. benthamiana* using agroinfiltration (Bozkurt et al., 2015) and compared them to coexpression with cytosolic free GFP (Figure 5). All three effectors produced a sharp fluorescent signal on the outside edge of the cytosol that overlapped with the YFP-Rem1.3 fluorescent signal and showed no detectable background in the cytosol (Figure 5) indicating that the three effectors accumulate at the pm when expressed in *N. benthamiana* cells. We also noted that the mCherry-PexRD31 mobile puncta reported above overlapped with the cytosolic GFP signal. In summary, we conclude that all three effectors accumulate at the pm, and that PexRD31 also localizes to mobile cytosolic bodies.

### PexRD31 localizes to RabC1-positive mobile bodies

**Figure 6:**
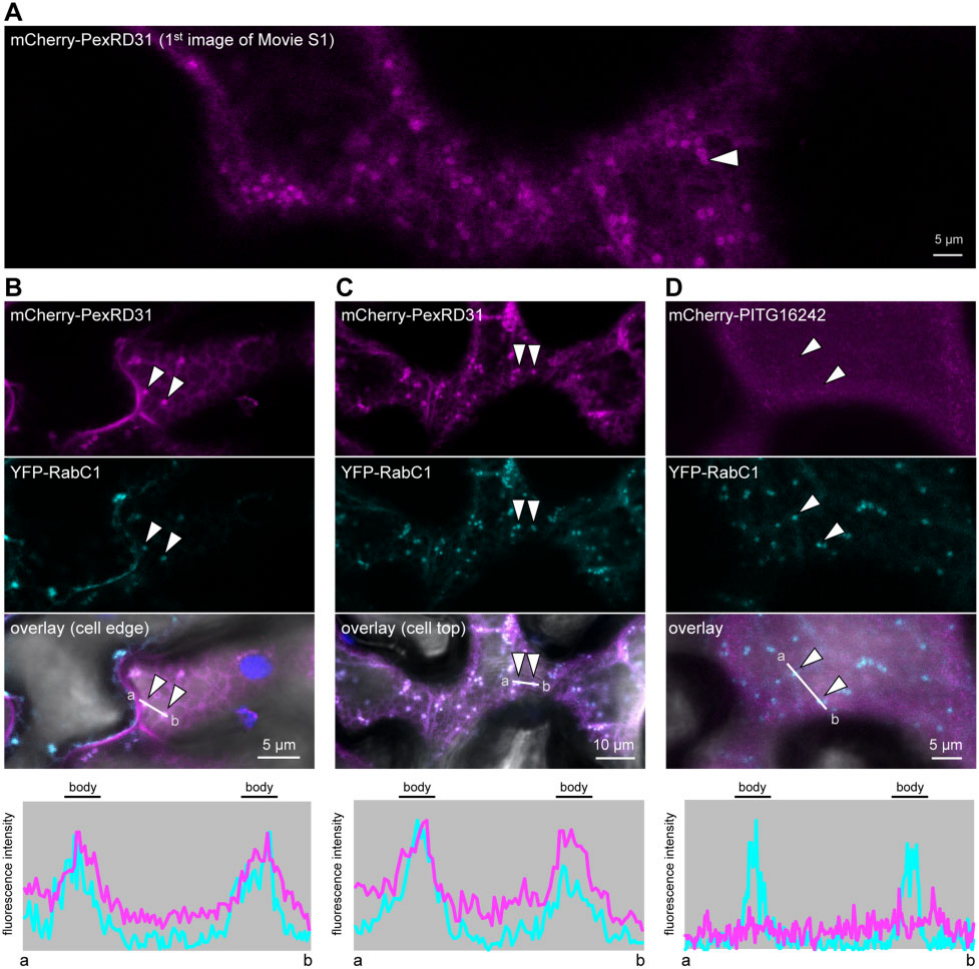
PexRD31 accumulates at RabC1-positive mobile bodies. A, Confocal microscopy image of *N. benthamiana* leaf epidermal cells expressing mCherry-PexRD31. The panel shows the mCherry channel. It corresponds to the first image of Supplemental Movie S1. B and C, Confocal microscopy images of the edge (B) and the top (C) of *N. benthamiana* leaf epidermal cells coexpressing mCherry-PexRD31 and YFP-RabC1. D, Confocal microscopy images of *N. benthamiana* leaf epidermal cells coexpressing mCherry-PITG16242 and YFP-RabC1. In (B–D), the lower panels show relative fluorescence intensity plots of the YFP (cyan) and the mCherry (magenta) along the line from a to b depicted in the corresponding overlay panel. In all cases, proteins were expressed in leaf cells by agroinfiltration. Live-cell imaging was performed with a laser-scanning confocal microscope 3 days after infiltration.

We further investigated the localization of PexRD31 cytosolic mobile puncta by coexpressing its GFP or mCherry fusions with a set of nine fluorescent proteins that mark different plant cell endomembrane compartments and organelles. These include secretory vesicles (VAMP724-mRFP), peroxisomes (GFP-PTS1), Golgi (MAN1_1-49_-GFP), phosphatidylinositol-3-phosphate (PI3P)-positive vesicles (2xFYVE-GFP), endosomes (mRFP-ARA7 and ARA6-mRFP), exocyst-positive organelle (Exo70E2-GFP), mitochondria (COX4_1-29_-GFP), and undefined mobile bodies (YFP-RabC1; Nelson et al., 2007; Voigt, 2008; Heard et al., 2015; Dagdas et al., 2016). Among these, we noted a sharp overlap in the fluorescent signals produced by mCherry-PexRD31 and YFP-RabC1 (Figure 6, B–C). These overlapping signals marked mobile puncta that are probably cytosolic vesicles (Geldner et al., 2009). These observations were specific to PexRD31 since another member of the PexRD12/31 family, PITG16242, did not colocalize with YFP-RabC1 (Figure 6D). The colocalization between PexRD31 and RabC1 was also specific in our experiments, as PexRD31 fluorescent signals did not overlap with any of the other markers with known subcellular localization (Supplemental Figure S7, A–H). We conclude that PexRD31 specifically localizes to RabC1-positive mobile bodies in addition to the pm.

### PexRD31 and NbVAMP72x coaccumulate at the pm

VAMP72x family in *A. thaliana* is known to localize at the plant cell pm (Uemura et al., 2004). Our results show that PexRD12/31 effectors also accumulate at the pm (Figure 5D–F). To determine whether NbVAMP72x and PexRD31 effector colocalize in plant cells, we transiently coexpressed RFP-tagged NbVAMP72x and GFP-tagged PexRD31 in *N. benthamiana* leaves by agroinfiltration (Bozkurt et al., 2015) and observed the fluorescence by live-imaging with a confocal microscope as described in the methods section. We found that the fluorescence signals from both proteins clearly overlap at the cell periphery which is likely to be at the pm (Supplemental Figure S7I). We conclude that both RFP-NbVAMP72x and GFP-PexRD31 accumulate in the same cellular compartment providing an opportunity for their association we observed using the coIP method in Figure 4.

### PexRD12/31 effectors accumulate at haustoria in *P. infestans*-infected *N. benthamiana* cells

**Figure 7:**
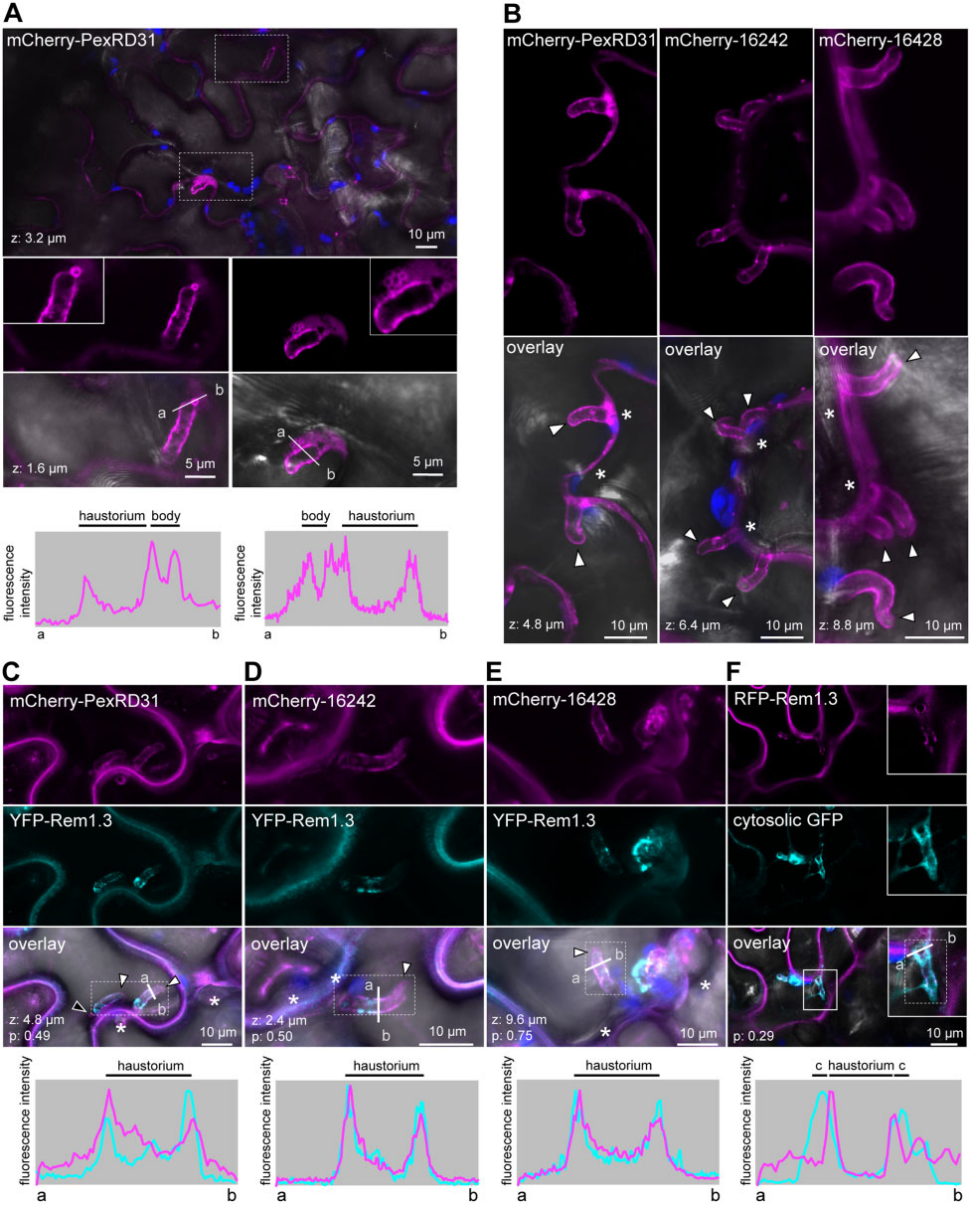
PexRD12/31 effectors accumulate around haustoria. A, Confocal microscopy images of *P. infestans* infected *N. benthamiana* leaf epidermal cells expressing mCherry-PexRD31. The top panel shows a low magnification overlay image; the two dotted-line rectangles indicate two haustoria from different cells that are shown at higher magnification in the images below. Inserts in the overlay images show a close-up of PexRD31-positive bodies associated with haustoria. The bottom panels show relative fluorescence intensity plots of mCherry along the line from a to b depicted in the corresponding overlay image. B, Confocal microscopy images of *P. infestans* infected *N. benthamiana* leaf epidermal cells expressing mCherry-PexRD31, mCherry-PITG16242, and mCherry-PITG16428 (top-left, top-right, and bottom-left, respectively). All fusions accumulate around haustoria. C–F, Confocal microscopy images of *P. infestans* infected *N. benthamiana* leaf epidermal cells coexpressing mCherry-PexRD31 (C), mCherry-PITG16242 (D), mCherry-PITG16428 (E), or cytosolic GFP (F) and PM/EHM marker YFP-Rem1.3. The black arrowhead indicates a PexRD31-labeled body in close proximity to the haustorium; white arrowheads indicate haustoria. Lower panels show relative fluorescence intensity plots of the YFP or GFP (cyan) and the mCherry (magenta) along the line from a to b depicted in the corresponding overlay images. c: cytosol. The insert in (F) shows a close-up of a haustorium. Dotted-line rectangles indicate the area considered to calculate the colocalization score (p; Pearson’s R-value) depicted at the bottom-left of overlay images. Images shown in this figure are single optical sections of 0.8 µm or maximal projections of up to 12 optical sections (max. z-stack of 9.6 µm). The overlay images combine YFP (C–E) or GFP (F), mCherry, chlorophyll, and bright-field images. White asterisks indicate extracellular hyphae, which can be out of focus. Fluorescent protein fusions were expressed (or coexpressed) in leaf cells by agroinfiltration, and leaves were drop inoculated with zoospores of *P. infestans* isolate 88069 3 h after agroinfiltration. Live-cell imaging was performed with a laser-scanning confocal microscope 3 days after infection.

We previously reported that several endomembrane compartments are directed toward *P. infestans* haustoria during infection (Bozkurt et al., 2012; Bozkurt et al., 2015; Dagdas et al., 2018; Bozkurt and Kamoun, 2020). In addition, some effectors display perihaustorial localization in infected plant cells (Bozkurt et al., 2011; Saunders et al., 2012; Dagdas et al., 2018; Wang et al., 2018). To investigate whether this applies to the PexRD12/31 family, we determined the subcellular localization of the mCherry fusions of PexRD31, PITG16242, and PITG16428 in haustoriated cells of *N. benthamiana* (Figure 7) using established protocols and methods (Bozkurt et al., 2011). All three proteins produced sharp fluorescent signals around haustoria indicating that they are perihaustorial effectors and accumulate at the haustorial interface when expressed in infected haustoriated plant cells (100% of haustoria imaged, *n* > 60; Figure 7A–B; Supplemental Movie S2).

In plants infected with *Phytophthora*, the host side of the haustorial interface comprises the EHM, a thin layer of cytosol, and the tonoplast that tends to be in close proximity to the EHM (Bozkurt et al., 2012). To further determine where the PexRD12/31 effectors accumulate, we coexpressed in *N. benthamiana* their mCherry fusions with YFP-Rem1.3, a marker that sharply labels EHM microdomains (Bozkurt et al., 2014 ). The fluorescent signal produced by the three effectors, but not by the cytosolic marker (used as negative control), sharply overlapped with the YFP-Rem1.3 signal, indicating that these effectors accumulate at the EHM (Figure 7C–F).

Experiments with *P. infestans*-infected *N. benthamiana* tissue enabled us to visualize the mCherry-PexRD31 puncta in haustoriated cells. Some of these puncta were immobile and were in proximity to the haustorial interface, enabling us to image them at higher resolution. This revealed that mCherry-PexRD31 produced a sharp circular fluorescent signal that can be observed near haustoria (*n* > 20l Figure 7A–C). We conclude that PexRD31 accumulates at vesicle-like structures and probably localizes on the outside of these vesicles. Consistent with our previous observations with non-infected *N. benthamiana* cells (Figures 5 and 6), PITG16242 and PITG16428 differed from PexRD31 and did not label vesicle-like structures. Altogether, these data indicate that PexRD31, PITG16242, and PITG16428 accumulate at the EHM around *P. infestans* haustoria. In addition, PexRD31 accumulates in vesicle-like structures in proximity to the haustorial interface.

### PexRD31 increases the number of FYVE-labeled endosomes in *N. benthamiana* cells

**Figure 8:**
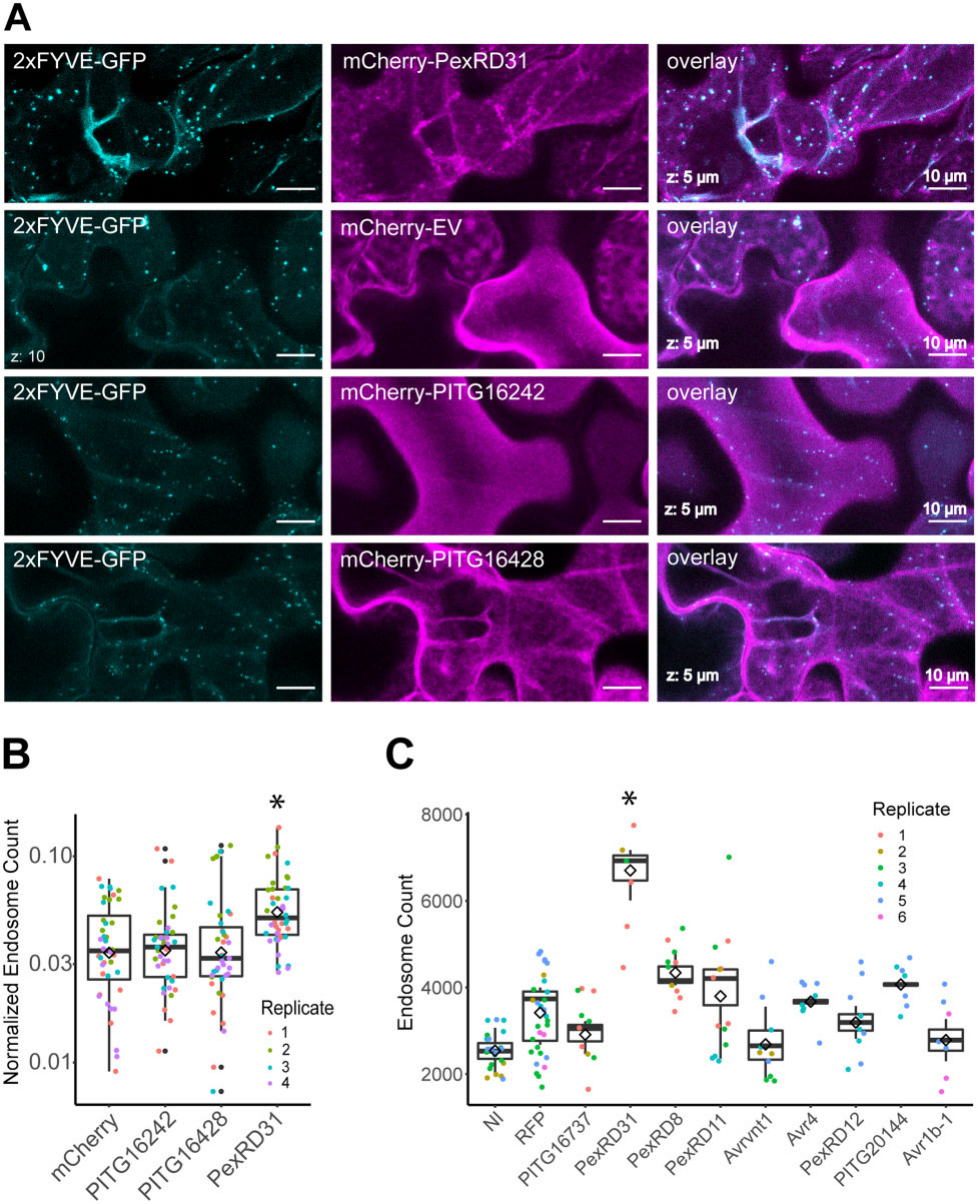
PexRD31 increases the number of FYVE labeled endosomes in *N. benthamiana* cells. A, Representative images of transient expression of mCherry-PexRD31, mCherry-EV, mCherry-PITG_16242, and mCherry-PITG_16428 in leaf epidermal cells of transgenic *N. benthamiana* lines expressing 2xFYVE-GFP. Scale bar = 10 µm. B, Categorical scatterplots with superimposed boxplots displaying the number of 2xFYVE-GFP endosomes µm^−2^ of cytoplasm in cells expressing mCherry-PexRD31, mCherry-PITG_16242, mCherry-PITG16428, or mCherry. The number of 2xFYVE-GFP-labeled endosomes was significantly enhanced by the expression of mCherry-PexRD31 (*P < 0.01, *n* = 40). The data are representative of maximum intensity projection images from four independent experiments performed on separate batches of plants. Each independent experiment consisted of five separate leaves, with 10 individual Z-stacks of 17 slices obtained per treatment (two per leaf). Images were taken 3 days post infiltration. C, Categorical scatterplots with super-imposed boxplots displaying the number of 2xFYVE-GFP endosomes per field of view in the presence of different FLAG-tagged effectors, RFP, or with no agroinfiltration (NI). 2xFYVE-GFP endosomes were significantly enhanced by the expression of PexRD31 (*P < 0.01, *n* = 6 for PexRD31 versus *n* = 24 for NI and *n* = 30 for RFP) with respect to the NI and RFP controls and the other effectors assayed. None of the other effectors assayed significantly increased the number of 2xFYVE-GFP-labeled endosome numbers. We performed between 2 and 4 independent experiments per treatment using separate batches of plants. Each independent experiment consisted of at least 2 individual z-stacks of 12 slices each per treatment obtained from 3 separate leaves. The data consist of averaged counts obtained from individual slices conforming each z-stack. Images were acquired 3 days post-infiltration.

During coexpression experiments with the FYVE marker (2xFYVE-GFP), we noted that PexRD31 appears to increase the number of GFP-labeled endosomes. We further investigated this observation by using agroinfiltration to express the mCherry fusions to PexRD31, PITG16242, and PITG16428 in leaves of transgenic *N. benthamiana* expressing 2xFYVE-GFP (Figure 8B–C). In independent experiments, mCherry-PexRD31 increased the number of FYVE-labeled endosomes by ∼40% compared to mCherry and the other two effectors (*n* = 40).

We also compared PexRD31 with nine *Phytophthora* RXLR effectors selected from our collection, which allowed to estimate the degree to which the observed activity is specific. These assays were done with a different expression vector system and experimental setup to determine the robustness of the effect. We performed agroinfiltration to express the FLAG-tagged effector constructs used earlier in the coIP experiments in the 2xFYVE-GFP transgenic *N. benthamiana* plants. PexRD31 significantly increased the number of 2xFYVE-GFP labeled endosomes, resulting in an increase of 40–90% compared to all other effectors and 90% compared to the negative control (Figure 8C). PexRD31 was the only effector to show a statistically significant increase with respect to the RFP control (*P* < 0.1, *n* = 6 for PexRD31, *n* = 30 for RFP). We conclude that PexRD31 specifically alters vesicle trafficking by increasing the number of PI3P endosomes labeled by the FYVE marker.

### FYVE-labeled endosomes aggregate in *N. benthamiana* tissue colonized by *P. infestans*

**Figure 9:**
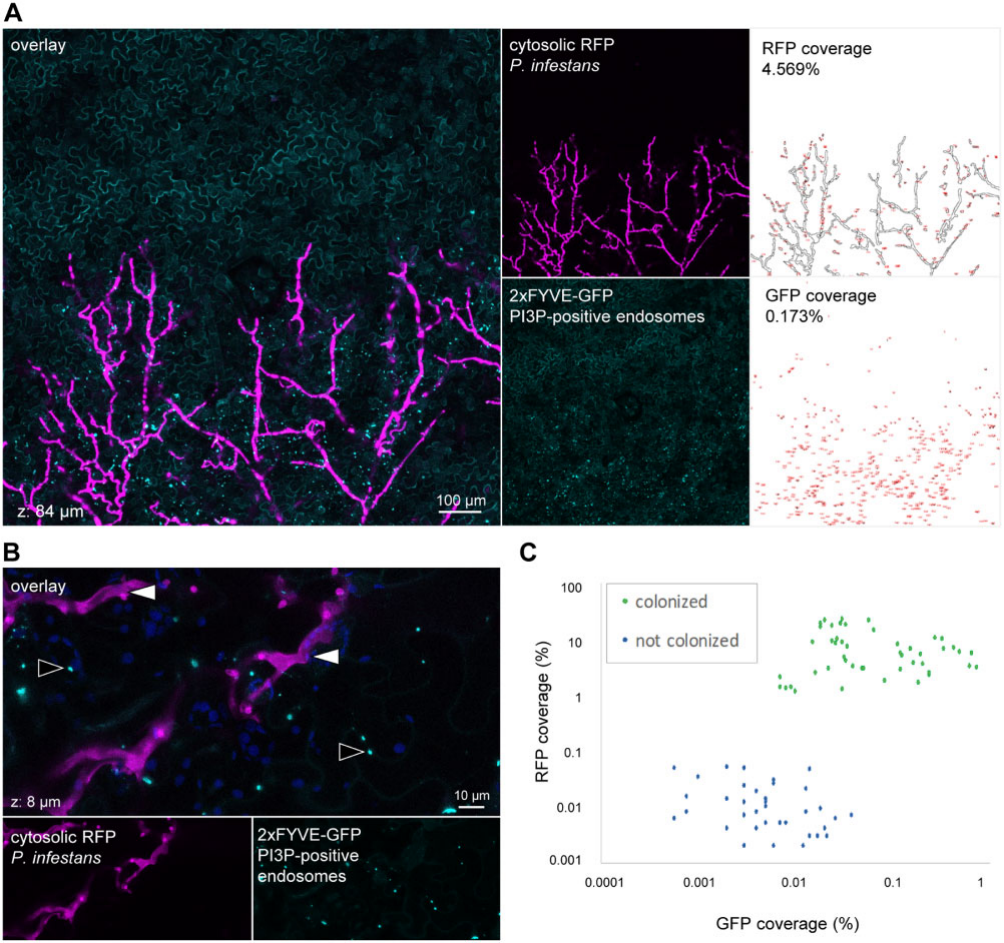
FYVE-labeled endosomes aggregate in leaf cells colonized by *P. infestans.* A, Low magnification live cell imaging of a 2xFYVE-GFP fusion (marker of PI3P-positive endosomes) in *N. benthamiana* leaf cells colonized by *P. infestans* isolate 88069td. The right-hand side panels show processed images used for the quantification of the image area displaying an RFP (upper panel) or a GFP puncta (lower panel) fluorescent signal, expressed as a percentage of the total image area. Images are maximal projections of 21 optical sections (z-stack of 84 µm). B, High magnification live cell imaging of a 2xFYVE-GFP fusion in *N. benthamiana* leaf cells colonized by *P. infestans* isolate 88069td. In the overlay image, black-lined white arrows indicate *P. infestans*, and white-lined black arrows indicate FYVE-labeled puncta. Images are maximal projections of 12 optical sections (z-stack of 9.6 µm). The image shown in the first image of Supplemental Movie S3. C, Categorical scatterplots with superimposed boxplots showing the semi-automated quantification of GFP puncta coverage from confocal microscopy images corresponding to leaf areas biotrophically colonized by *P. infestans* (“Colonized”) or leaf areas without *P. infestans* (Not Colonized; ^*^P < 0.01). Leaves of stable transgenic *N. benthamiana* plants were drop inoculated by zoospores of *P. infestans* isolate 88069td. Live-cell imaging was performed with a laser-scanning confocal microscope 3 days after inoculation. The overlay panels combine (A) GFP and RFP channels, or (B) GFP, RFP, and chlorophyll channels.

To determine whether the effect of PexRD31 on FYVE-labeled endosomes can be recapitulated in the presence of the pathogen, we examined the number and distribution of FYVE-labeled endosomes during *P. infestans* infection of *N. benthamiana* leaves. To this end, we inoculated *N. benthamiana* plants expressing 2xFYVE-GFP with *P. infestans* 88069td, a transgenic strain expressing the red fluorescent marker tandem dimer RFP (tdTomato; Whisson et al., 2007; Giannakopoulou et al., 2014). We evaluated 2xFYVE-GFP fluorescence signals relative to the RFP fluorescence and focused our imaging on the edge of the disease lesions where haustoria are produced and which corresponds to the biotrophic phase of infection (van West et al., 1998; Lee and Rose, 2010). Using low magnification confocal microscopy, we noted that leaf areas biotrophically colonized by *P. infestans* display bright and large GFP puncta; such puncta were absent in non-colonized areas (Figure 9A). *Nicotiana benthamiana* cells with GFP puncta signals were in direct contact with *P. infestans* hyphae, whereas the non-colonized areas surrounding the lesions showed hardly any GFP puncta signals. 2xFYVE-GFP puncta were mobile, with a maximal diameter of 2 µm (Figure 9B;Supplemental Movie S3). We compared these patterns to *P. infestans* infections of *N. benthamiana* stable transgenics expressing free GFP (marking the nucleus and the cytosol) or a YFP-RabG3c fusion (marking late endosomes and the tonoplast). In both cases, there was no evident alteration of the distribution of the fluorescence signals in tissue colonized by *P. infestans* (Supplemental Figure S8), indicating that *P. infestans* specifically perturbs 2xFYVE-GFP-positive compartments during the biotrophic colonization of *N. benthamiana*.

**Figure:**
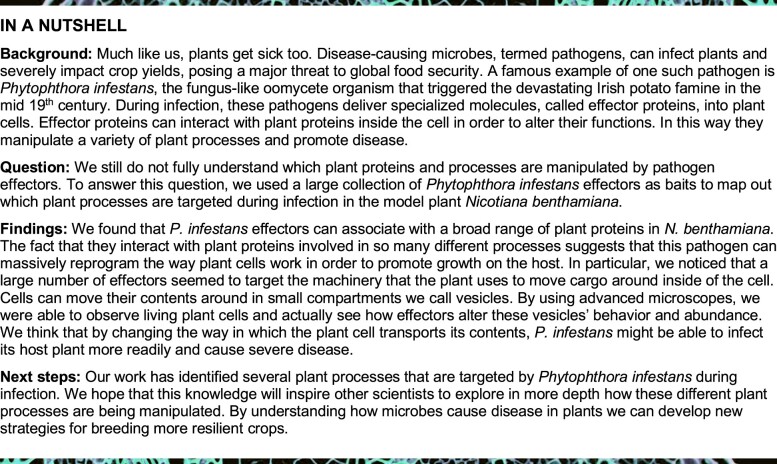


To further evaluate the correlation between the presence of *P. infestans* hyphae and the formation of FYVE-labeled puncta, we performed a blind confocal microscopy image acquisition analysis. We acquired 13 z-stacks of 11 images (*n* = 11) over leaf tissues colonized by *P. infestans* 88069td and surrounding non-colonized areas. Next, we developed and applied an automatic analytical pipeline using the ImageJ package Fiji (Schindelin et al., 2012) to quantify *P. infestans* red fluorescence and punctate GFP-FYVE fluorescence signals. This experiment further showed that 2xFYVE-GFP-positive puncta signals increased in *N. benthamiana* tissue during the biotrophic stage of *P. infestans* infection compared to the non-colonized areas of leaf tissue (Figure 9C). This set of experiments indicate that *P. infestans* alters host endosome trafficking during biotrophic colonization.

## Discussion

A major research aim in the field of molecular plant pathology is to unravel the activities of effectors in order to understand how pathogens successfully colonize their hosts. The rationale behind this project is based on the view that effectors can serve as molecular probes to identify the host processes that pertain to pathogen interactions. We applied an effectoromics pipeline centered on an in vivo proteomics-based protein–protein interaction screen. This screen revealed that ∼50 *P. infestans* RXLR effectors associate in planta with ∼580 unique *N. benthamiana* proteins representing as many as 35 biological processes. We conclude that *P. infestans* RXLR effectors target multiple processes in their host plants; and our study provides a broad overview of these effector-targeted processes.

We previously reported in planta proteomics screens for interactors of candidate effectors of the rust fungal pathogens *Melampsora larici-populina* and *Puccinia striiformis* f sp *tritici* (Petre et al., 2015; Petre et al., 2016). Our study also complements large-scale yeast-two-hybrid screens for effector interactors of the oomycete plant pathogen *Hyaloperonospora arabidopsidis*, the bacterium *Pseudomonas syringae* and the powdery mildew fungus *Golovinomyces orontii* (Mukhtar et al., 2011; Weßling et al., 2014). As previously discussed (Petre et al., 2015; Petre et al., 2016), in planta protein–protein interaction assays have both advantages and disadvantages compared to the more commonly used yeast-two hybrid assay. Given that the coIP assay takes place in vivo in plant tissue, the host proteins are expressed in a biologically relevant molecular context and cellular compartment. Another difference is that yeast two-hybrid interactions are presumably binary, detecting one-on-one protein interactions, whereas the proteins we identified by coIP/MS may not directly bind the effector but could instead associate in a multi-protein complex. Indeed, our observation that functionally related plant proteins tend to associate with a given effector family increases the probability that the targeted host complex or process is physiologically relevant (Figure 2).

One drawback of the coIP method is that a direct host interactor may be missed even when relevant associated proteins are recovered. This could be due to several factors, such as abundance of the interacting plant proteins, absence of a suitable tryptic peptide detectable by MS, or absence of the protein sequence in the annotated database used for the mass spectra searches. Also, given that MS sampling of peptides is random, a target protein may be missed by chance. One example is the *P. infestans* RXLR-WY effector PexRD54, which binds with high affinity to the autophagy protein ATG8CL (Dagdas et al., 2016; Maqbool et al., 2016). Although ATG8CL was missing from the initial coIP/MS screen it was picked up in two out of four subsequent experiments (Dagdas et al., 2016). This might be due to the low molecular weight of ATG8CL (∼14 kDa), which may not have produced enough peptides for MS detection. Interestingly, we flagged ATG8C as likely to directly bind PexRD54 because their interaction survived stringent binding conditions in contrast to other candidate interactors (Dagdas et al., 2016). Rab8a, another interactor identified in the original PexRD54 coIP/MS experiment, was recently reported as a genuine target of PexRD54 that associates with this effector independently of ATG8CL binding (Pandey et al., 2020). The PexRD54 work illustrates the importance of replication and follow-up experiments to the coIP/MS. As with any biochemical assay, there is a benefit to experiment with different assay conditions, such as binding stringency.

A subset of the host proteins we identified can be tagged as “usual suspects” that are unlikely to form biologically relevant interactions with the effectors (Petre et al., 2015; Petre et al., 2016). Nonetheless, 497 of the host interactors associated with no >4 out of the 14 effector families (Supplemental Data Set S2). These relatively specific interactors are more likely to be biologically relevant compared to the more promiscuous proteins, which could include genuine “hubs” but are probably enriched in false positives (Petre et al., 2015; Petre et al., 2016). In total, six of the interactions we identified have been reported and analyzed in earlier studies (Supplemental Table S1; Figure 1C). Indeed, as our follow-up analyses of the PexRD12/31 effector family demonstrate, the interactome network we generated can serve as a launchpad for studies of effector activities and the perturbations they cause in host plant cells. We, therefore, hope that this interactome dataset will complement previous screens of *P. infestans* RXLR effectors aimed at studying effector localization in the host and effector activity in suppressing pattern-triggered immunity (Zheng et al., 2014; Wang et al., 2018) and serve as a useful resource for functional effector biology studies.

Classification of the host interactors into putative functional categories revealed over 35 biological processes that are candidate effector-targeted processes (Supplemental Table S2). The diversity of these processes is consistent with the view that *Phytophthora* RXLR effectors have evolved to modulate host pathways as diverse as immune signaling, gene silencing, and selective autophagy (Bos et al., 2010; Qiao et al., 2015; Dagdas et al., 2016). However, the interactome network also illustrates a degree of convergent targeting of particular processes both within and between RXLR effector families. Indeed, even though effectors can converge on a few “hub” host proteins (Song et al., 2009; Mukhtar et al., 2011), one challenge is to understand how multiple effectors act on different steps of a host-targeted pathway (Win et al., 2012b). A classic example of this concept can be observed in plant viruses, which encode several proteins that have evolved different mechanisms to suppress host RNA silencing rather than converging on a single host molecule (Burgyán and Havelda, 2011). In another example, *P. infestans* counteract apoplastic cysteine proteases by inhibiting host protease secretion via the RXLR effector AVRblb2 and by secreting protease inhibitors in the apoplast (Tian et al., 2007; Bozkurt et al., 2011). Indeed, the architecture of the interactome we generated indicates a certain degree of convergence between unrelated *P. infestans* effectors toward certain host processes, for example, PexRD12/31 family and Avr2 family showed associations with common proteins involved in vesicle trafficking pathway. However, there is no experimental evidence of perturbation by Avr2 family to vesicle trafficking pathway.

In light of the differences between the protein–protein interaction methods described above, it is relevant to note that we recovered relatively few transcription factors or nuclear proteins compared to yeast two-hybrid screens. This could be explained by the protein extraction protocol we used, which may have yielded a proteome relatively depleted in nuclear proteins (Howden and Huitema, 2012). On the other hand, our interactome was enriched in vesicle trafficking proteins possibly reflecting the value of having the effector baits expressed in planta in a cellular context that more closely mirrors dynamic cellular processes, such as endomembrane trafficking.

During *P. infestans* infection, RXLR effectors are thought to be delivered inside haustoriated cells where they orchestrate cellular and molecular reprogramming of these plant cells, notably by modulating membrane trafficking (Bozkurt et al., 2012; Dagdas et al., 2016; Dagdas et al., 2018). However, the mechanisms by which *P. infestans* RXLR effectors and other plant pathogen effectors block or hijack host membrane trafficking remain poorly understood. Several RXLR effectors, such as *P. infestans* AVRblb2 and AVR1, *Phytophthora palmivora* REX3, and *Phytophthora brassicae* RxLR24, prevent the secretion of host proteins presumably to counteract focal immune responses in the host plant (Bozkurt et al., 2011; Du et al., 2015; Evangelisti et al., 2017; Tomczynska et al., 2018). Another *P. infestans* effector, PexRD54, stimulates and co-opts plant membrane trafficking by associating with the plant autophagy machinery at polarized foci in the haustorial interface (Dagdas et al., 2018). PexRD54 was also recently proposed to connect small GTPase Rab8a vesicles with the autophagy machinery to stimulate formation of autophagosomes at the pathogen interface (Pandey et al., 2020). In our host-interactor screen, we found that ten effector families associated with 32 proteins implicated in vesicle mediated transport (Figure 2; Supplemental Figure S3; Supplemental Table S2). These results, along with our follow-up observation that members of the PexRD12/31 family associate with R-SNARE VAMP72 proteins and accumulate at the haustorial interface, further point to vesicular trafficking as a major target of oomycete effectors.

PexRD12/31 effectors add to a growing list of perihaustorial effectors that are associated with polarized markers at the *P. infestans* haustorial interface (Bozkurt and Kamoun, 2020). We also discovered that the PexRD12/31 family of RXLR-WY effectors associate with 16 vesicle-trafficking *N. benthamiana* proteins in a relatively specific manner (Supplemental Table S3). However, at this stage, we did not determine which of these host proteins, including NbVAMP72x, directly bind the effectors. It remains possible that the PexRD12/31 effectors have the same direct host interactor and that the additional proteins discovered by coIP/MS are part of a multiprotein complex targeted by these effectors. Nevertheless, PexRD12/31 effectors accumulate at the host pm and, therefore, could potentially colocalize with vesicle trafficking components at this subcellular location. In addition, the examined effectors focally accumulate at the EHM suggesting that they are associated with the dynamic membrane trafficking processes that take place in the infected haustorial cells. PexRD31 stood out compared to the other examined effectors by labeling mobile vesicle-like puncta; and these PexRD31 vesicle-like structures were visualized in contact with the EHM (Figures 7–9). PexRD31 colocalized with RabC1, but, unfortunately, the biology of RabC1 and the exact nature of RabC1-positive bodies is unknown (Geldner et al., 2009). Recently, VAPYRIN, a protein required for the intracellular establishment of arbuscular mycorrhizal fungi, was shown to colocalize with RabC1 in small mobile structures (Bapaume et al., 2019). Interestingly, VAPYRIN also interacts with a symbiotic R-SNARE of the VAMP72 family (Bapaume et al., 2019). Therefore, VAPYRIN/RabC1 mobile bodies might mark an endosomal pathway associated with perimicrobial membranes of plant cells colonized by filamentous microbes. Future studies will determine whether the PexRD31 and VAPYRIN bodies form another example of common cellular structures formed by pathogens and symbionts during intracellular colonization of plant cells (Rey and Schornack, 2013).

We found that PexRD31 and *P. infestans* alter the number and the distribution of FYVE-labeled endosomes in leaves of *N. benthamiana*. How would a pathogen benefit by modulating FYVE-positive endosomes? The 2xFYVE-GFP marker labels vesicles enriched in the phosphoinositide lipid PI3P, notably endosomes and multivesicular bodies of the endocytic pathway (Robinson et al., 2008; Gao et al., 2014). Our finding that *P. infestans* alters PI3P vesicles is consistent with previous reports that this pathogen perturbs plant endocytic trafficking, notably by redirecting this pathway to the EHM (Bozkurt et al., 2015). Pathogen-induced modifications in host membrane phosphoinositide composition have recently been proposed both as a potential pathogen strategy to promote infection and as a host focal immune response (Rausche et al., 2020). *Phytophthora infestans* may deploy effectors such as PexRD31 to boost the late endocytic pathway, help recruit endomembranes for EHM biogenesis and counter host immunity. Another possibility is that PexRD31 blocks PI3P vesicle fusion to host membranes resulting in the accumulation of these vesicles in the cytosol. Further mechanistic studies of the dynamics of the late endocytic pathway during *P. infestans* infection should reveal the biological significance of the observed phenomenon.

*Phytophthora infestans* is an aggressive plant pathogen that continues to threaten global food security (Kamoun et al., 2015). Our study adds to the in planta effectoromics screens that have been conducted with *P. infestans* RXLR effectors ever since the sequencing of this pathogen’s genome over 10 years ago (Haas et al., 2009; Oh et al., 2009; Pais et al., 2013; Zheng et al., 2014; Wang et al., 2018). To date, high-throughput screens have focused primarily on assigning AVR activities to effectors, which helped guide the identification and characterization of matching plant immune receptors (Vleeshouwers et al., 2008; Oh et al., 2009; Rietman, 2011). The interactome network resource we have generated complements these earlier screens and should prove to be a valuable platform for functional studies of *P. infestans* effectors and the processes they target.

## Materials and methods

### Biological materials

We used *Escherichia coli* strain DH5α, *A. tumefaciens* strain GV3101 (pMP90), and *N. benthamiana* as previously described (Petre et al., 2015). *Nicotiana benthamiana* seeds were germinated on germinating medium, transplanted individually into 9 cm pots containing peat-based composted soil, and grown under controlled growth conditions of 20–22°C, average humidity of 60%, and 16/8 h light/dark cycle under fluorescent bulbs (Daylight, 6200 K) with a photosynthetic photon flux density of 150 µmol m^−2^ s^−1^. Transgenic 2xFYVE-GFP *N. benthamiana* were obtained by transforming the plants with binary plasmid construct pBLTI221-2xFYVE-GFP (Voigt et al., 2005). We made a single transformation of *N. benthamiana* with the construct and propagated the transformants to T2 generation. We selected a highly expressing stable line based on the observation with confocal microscopy for further experiments. *Phytophthora infestans* isolates 88069 and 88069td, expressing the red fluorescent marker tandem dimer RFP (tdTomato), were grown and used for *N. benthamiana* leaf infection as previously reported (Dagdas et al., 2016).

### Plasmid construction

Molecular cloning and recombinant DNA manipulations were conducted using standard protocols. Primers and coding sequences of the fusion proteins used in this study are indicated in Supplemental Table S4 and Supplemental Data Set S5, respectively.

To generate FLAG-tagged protein fusions, we obtained DNA fragments matching the coding sequence of the effector domains by gene synthesis (GenScript, Piscataway, NJ, USA) including *Pac*I and *Not*I restriction sites on 5′ and 3′ ends, respectively (Supplemental Data Set S5). Through this process, the coding sequences were codon-optimized for expression in *N. benthamiana* and the N-termini (signal peptide and RXLR regions) were replaced by FLAG tag sequences. We then cloned the DNA fragments into the Tobacco mosaic virus-based *A. tumefaciens* binary vector pTRBO (Lindbo, 2007).

To generate fluorescent protein fusions, we obtained the coding sequence of effector domains by PCR amplification from genomic DNA of *P. infestans* isolate T30-4, using primers that included *Bbs*I AATG/GCTT-compatible sites. DNA fragments were cloned into the Golden Gate level 0 vector pICSL41308 by digestion/ligation as previously described (Petre et al., 2015; Petre et al., 2017) and verified by sequencing (GATC Biotech, Constance, Germany). DNA fragments were combined into a Golden Gate level 1 binary vector pICSL47742 in order to create a “CaMV 35S promoter:fluorescent protein coding sequence:effector domain coding sequence:OCS terminator” expression unit.

The following fluorescent markers were described previously: mitochondria (COX4_1-29_-GFP), peroxisomes (GFP-PTS1), Golgi apparatus (MAN1_1-49_-GFP), and ER (WAK2_SP_-GFP-HDEL; Nelson et al., 2007); PI3P-positive vesicles (2xFYVE-GFP; Voigt, 2008). We synthesized the coding sequence of *A. thaliana* Exo70E2 (At5g61010; GenScript) and assembled it with fluorescent proteins into plasmid vector pICH86988 as previously described (Petre et al., 2015).

GFP-NbVAMP72x was cloned using Gateway technology (Thermo Fisher Scientific, Waltham, MA, USA) as follows. Coding sequence for NbVAMP72x was amplified by PCR using the primer pair NbVAMP72x_F and NbVAMP72x_R (Supplemental Table S4), and Pfu taq polymerase (Takara, Mountain View, CA, USA) from *N. benthamiana* cDNA made from total RNA extracted from 4-week-old *N. benthamiana* leaves. PCR was performed for 35 cycles with denaturing at 96°C for 30 s, annealing at 58°C for 40 s and extension at 72°C for 1 min for each cycle, followed by final extension at 72°C for 10 min. The amplicon was cloned into pENTR (Thermo Fisher) using a Topo TA cloning kit (Thermo Fisher). The insert was transferred to the destination vector pK7WGF2 by LR reaction using Gateway LR Clonase II Enzyme mix (Thermo Fisher) in frame with the coding sequence of GFP which was part of the vector. All Gateway cloning was performed following the manufacturer’s instructions.

### Immunoblotting

We performed immunoblot analyses on SDS-PAGE separated proteins as described elsewhere (Oh et al., 2009). We used Monoclonal FLAG M2-alkaline phosphatase antibodies (Sigma-Aldrich, St-Louis, MO, USA) at a 1:10,000 dilution and we developed blots using the AP conjugate substrate kit (Bio-Rad; Hercules, CA, USA). We used polyclonal GFP antibodies (Thermo Fisher) at a 1:4,000 dilution as primary antibody, and anti-rabbit polyclonal antibody conjugated to horseradish peroxidase (Sigma-Aldrich, St-Louis, MO, USA) as a secondary antibody at a 1:12,000 dilution. We detected protein band signals using ECL substrate (Thermo Fisher) following exposure on Amersham Hyperfilm ECL (GE Healthcare, Chicago, IL, USA).

### coIP assays

We performed anti-FLAG coIP/MS assays following a protocol previously described (Win et al., 2011). Briefly, the strategy consisted of affinity purifying transiently expressed FLAG epitope-tagged effector proteins and identifying the plant proteins associated with purified effectors by MS (Supplemental Figure S1). Agrobacteria containing each effector construct were infiltrated into third and fourth leaves of 4–8 week-old *N. benthamiana* plants at 0.5 OD_600_ concentration to transiently express the FLAG-tagged effectors. Leaves were harvested 3 days after infiltration. FLAG-tagged effector proteins were immunoprecipitated using agarose beads conjugated with anti-FLAG monoclonal antibodies. Bound effectors and associated plant proteins were competitively eluted by 3XFLAG peptides (Sigma-Aldrich, Dorsett, England) and separated by SDS-PAGE. Each lane from the gel was cut into strips, proteins were digested in gel with trypsin, and submitted for protein identification by MS. For spectral searches, we used a protein sequence database composed of two proteome predictions of *N. benthamiana* genome (Bombarely et al., 2012): (1) proteome version 0.44 available at the SGN (http://solgenomics.net/) and (2) evidence-based proteome prediction made by The Genome Analysis Center based on the same genome sequence. Identical sequences were removed from the combined database to create a non-redundant *N. benthamiana* proteome sequence database. Each sequence in the database was annotated with a top BLASTP hit (e-value cutoff 1 × 10^−3^) to SwissProt protein database if the search was successful. This annotated database was used for Mascot searches using the spectral collection from the MS. Mascot results were analyzed using Scaffold (Proteome Software Inc., Portland, OR, USA). Presence of a protein in the analyzed samples was identified by having at least two peptide matches with ≥95% probability and an ion score of equal or >40 in their matches. All protein hits fulfilling these criteria were exported from Scaffold program for further analysis. To reduce complexity of the dataset, we grouped the RXLR effectors into families as previously described (Boutemy et al., 2011) based on their effector domains using a Markov clustering algorithm (Enright et al., 2002). To further reduce the complexity of the dataset, host protein interactors were also clustered if they shared (1) a minimum of 80% identity on 80% of their lengths, or (2) the exact same annotation. Each cluster was represented by a single identifier for downstream analyses. Each plant protein identified in coIP/MS was annotated with GO terms using Blast2Go program (Götz et al., 2008).

For a subset of samples, we performed anti-GFP coIP/MS assays and subsequent analyses as previously reported (Win et al., 2011; Petre et al., 2015; Petre et al., 2017), using GFP-Trap agarose beads (Chromotek, Munich, Germany), a hybrid mass spectrometer LTQ-Orbitrap XL (Thermo Fisher Scientific, Carlsbad, CA, USA) and a nanoflow-UHPLC system (NanoAcquity Waters Corp., Burnsville, MN, USA). However, to be more stringent during the IP process, we used extraction and IP buffers with 0.5% IGEPAL and 400 mM NaCl. We performed two technical replicates for the trypsin digestion. For the first replicate, we followed the in-gel digestion protocol as previously reported (Petre et al., 2015). For the second replicate, we performed on-bead digestion as described previously (Zess et al., 2019). Both methods yielded similar results (Supplemental Figure S9; Supplemental Data Set S3). We merged the total spectrum count values from the two technical replicates for the analyses shown in Supplemental Table S3.

### Phylogenetic analyses

We obtained amino acid sequences of the PexRD12/31 family effectors from GenBank based on “RxlRfam9” RXLR family reported in Haas et al. (2009). We removed identical sequences and pseudogenes from the family and collected 16 sequences in a database for further analysis (Figure 3). We used effector domain sequences (after “EER” residues, Figure 3) for multiple sequence alignment by MAFFT program using “–auto –reorder” options and L-INS-I strategy (Katoh et al., 2005). Multiple sequence alignment was performed with iterative refinement method (<16) with local pairwise alignment information using amino acid substitution matrix BLOSUM62, 1.53, with the amino acids colored according to the ClustalX scheme (Larkin et al., 2007). The phylogenetic relationship within the PexRD12/31 family members was inferred using the Neighbor-Joining method implemented in MEGA X (Kumar et al., 2018) with 100 bootstraps. Sequence conservation profile was obtained using the WebLogo server (http://weblogo.berkeley.edu; Crooks et al., 2004). Secondary structure prediction was done using Jpred server (http://www.compbio.dundee.ac.uk/jpred; Drozdetskiy et al., 2015). WY-fold region was predicted based on the hidden Markov model (HMM) reported in Boutemy et al. (2011). HMMER version 3.1b (Mistry et al., 2013) was used to search the WY-fold HMM in amino acid sequences of PexRD12/31 family of effectors.

To derive a phylogeny for VAMP proteins, the sequence of NbS00022342g0004 was searched against the *A. thaliana* proteome version Araport11 (The Arabidopsis Information Resource (TAIR), https://www.arabidopsis.org) using BLASTP (BLAST 2.9.0+; Altschul et al., 1997) as provided by TAIR server. To attempt to identify an ortholog of NbS00022342g0004 in *A. thaliana*, we extracted all AtVAMP72 family (AtVAMP721-727) from Araport11 and 14 VAMP72-like amino acid sequences from the proteome of *N. benthamiana* (SGN, version 0.44). Multiple Sequence alignment was performed with MAFFT program using “–auto –reorder” options and L-INS-I strategy (Katoh et al., 2005). Phylogeny was inferred by using the Maximum Likelihood method and JTT matrix-based model (Jones et al., 1992) implemented in MEGA X (Kumar et al., 2018). Initial tree(s) for the heuristic search were obtained automatically by applying Neighbor-Join and BioNJ algorithms to a matrix of pairwise distances estimated using the JTT model, and then selecting the topology with superior log likelihood value after 1,000 bootstraps.

### Laser-scanning confocal microscopy

We used laser-scanning confocal microscopy to observe the fluorescence-fusion proteins in *N. benthamiana* leaves after transiently expressing the proteins by agroinfiltration. We infiltrated at least three leaves per construct on 2–3 plants that are 4–6 weeks old in each experiment and repeated the experiment at least 3 times on different days with different batches of plants. We collected the leaves 3 days post-agroinfiltration and immediately performed live-cell imaging with a Leica DM6000B/TCS SP5 laser-scanning confocal microscope (Leica microsystems, Bucks, UK), using a 63× water immersion objective as previously described (Petre et al., 2015). More than 10 fields of views were analyzed per experiment. We used the following settings for excitation/collection of fluorescence: GFP (488/505–525 nm), chlorophyll (488/680–700 nm), and mCherry (561/580–620 nm). We performed image analysis with Fiji (http://fiji.sc/Fiji).

### Quantification of FYVE endosomes in transient assays

To quantify the 2xFYVE-GFP puncta in confocal images acquired from *N. benthamiana* leaf cells transiently expressing different mCherry or RFP-fused constructs, we built a pipeline using the ImageJ package Fiji (Schindelin et al., 2012). We split the channels (GFP, RFP, and bright field) and considered only the GFP channel. We then smoothed the images with the Smooth tool. Third, we performed an auto threshold using the RenyiEntropy parameter. Finally, we counted the puncta per image with the Analyze particles tool, with the following settings: size = 0.07–5; circularity = 0.3–1. In parallel, we calculated an estimate of the area of cytoplasm using the total GFP signal in the image. To do this, we first applied the Gaussian blur tool with a Sigma = 5. We then applied the Threshold function using the Huang white method. After that, we obtained the area of cytoplasm using the Measure tool. The number of GFP puncta calculated in every image was divided by the total area of cytoplasm calculated, obtaining the number of GFP labeled puncta µm^−2^ of cytoplasm. We then exported the quantification results in a tab-separated text file. The data were analyzed using Tukey’s multiple comparisons test using the linear model in R (R Core Team, 2020), with differences being considered significant when *P* < 0.01.

For the quantification of 2xFYVE-GFP puncta in confocal images acquired from *N. benthamiana* leaf cells transiently expressing FLAG-tagged *Phytophthora* effectors and free RFP, a different automated pipeline described in (Salomon et al., 2010) was employed. The data were analyzed using Tukey’s multiple comparisons test using the linear mixed-effects model in R (R Core Team, 2020) due to variations in number of points being analyzed, with differences being considered significant when *P* < 0.01.

### Quantitative analysis of confocal images during *P. infestans* leaf colonization

To quantify the 2xFYVE-GFP puncta in confocal images acquired on *N. benthamiana* leaf cells colonized by *P. infestans* isolate 88069td, we built a four-step analytical pipeline using the ImageJ package Fiji (Schindelin et al., 2012). First, we split the channels (GFP, RFP, chlorophyll, and bright field) and considered only the GFP channel. Second, we smoothed the images with the Smooth tool. Third, we performed an auto threshold using the RenyiEntropy parameter. Finally, we counted the puncta per image with the Analyze particles tool, with the following settings: size = 0.5 – infinity; circularity = 0.5 – 1. To quantify the presence of *P. infestans* isolate 88069td on *N. benthamiana* leaves, we adapted the first and last steps of the pipeline abovementioned as follow: for the first step, we considered the RFP channel, and for the fourth step, we used the default settings for particle size and circularity, that is, 0 – infinity and 0–1, respectively. We then exported the quantification results in a spreadsheet and generated the scatterplot using R (R Core Team, 2020).

### Quantitative analysis of colocalization assays

To quantify fluorescent signal overlap (colocalization) in discrete objects shown in confocal microscopy images, we used fluorescence intensity plots. To this end, we first used the plot profile tool in Fiji to quantify pixel intensity along a line for each channel (cyan and magenta). Second, we exported the data directly into a spreadsheet and normalized the dataset based on their maximal values. Finally, we built the relative intensity plots shown in the figures using the Microsoft Excel 2-D line tool, and annotated them in Microsoft PowerPoint.

To calculate colocalization scores shown in Figures 5 and 7, we used the coloc2 tool in Fiji (which allows to quantify pixel intensity correlation over space), using default parameters. We considered the Pearson’s R-value (no threshold) in the relevant images and indicated it in relevant images (p).

### Data availability and accession numbers

GenBank accession numbers for the effectors used in this study are presented in Table 1. The Solanaceae Genomics Network (https://solgenomics.net) accession numbers for *N. benthamiana* proteins are listed in Supplemental Data Set S1A and the amino acid sequences are provided in Supplemental Data Set S1B. Machine-readable phylogenetic tree files for PexRD12/31family (depicted in Figure 3) and NbVAMP family (depicted in Supplemental Figure S5) are provided in Newick format in Supplemental Files S1 and S2, respectively.

An interactive version of the effector-plant protein network depicted in Figure 2 is publicly available at The Network Data Exchange repository with unique ID d60890f4-5c95-11eb-9e72-0ac135e8bacf (http://public.ndexbio.org/viewer/networks/d60890f4-5c95-11eb-9e72-0ac135e8bacf)

The MS proteomics data were deposited to the ProteomeXchange Consortium via the PRIDE (Perez-Riverol et al., 2019) partner repository (https://www.ebi.ac.uk/pride/archive) with the dataset identifier PXD020751 and 10.6019/PXD020751.

The raw data that pertain to microscopy assays (raw images and colocalization analyses reports) and phylogenetic tree construction are available in Zenodo repository with DOI 10.5281/zenodo.4495248 (https://doi.org/10.5281/zenodo.4497844).

## Supplemental data

The following materials are available in the online version of this article.

**Supplemental Figure S1.** Strategy to screen for effector-associated plant proteins in *N. benthamiana.*

**Supplemental Figure S2.** Anti-FLAG IP efficiently purifies the effector fusions.

**Supplemental Figure S3.** Subnetworks of the RXLR effector interactome organized by GO terms.

**Supplemental Figure S4.** Anti-GFP coIP efficiently purifies GFP-tagged fusions transiently expressed in *N. benthamiana* leaves.

**Supplemental Figure S5.** NbVAMPs associated with PexRD12/31 effectors belong to the VAMP72 family in *N. benthamiana.*

**Supplemental Figure S6.** PexRD12/31 effectors accumulate mainly at the cell periphery and around haustoria during *P. infestans* infection.

**Supplemental Figure S7.** PexRD31 does not accumulate in the main cell compartments but coaccumulates with NbVAMP72x at the pm.

**Supplemental Figure S8.** *Phytophthora infestans* biotrophic colonization does not trigger the formation of punctate signals in the nucleocytosolic, late endosomal, and tonoplast compartments.

**Supplemental Figure S9.** In-gel and on-bead trypsin digestion methods yield similar data.

**Supplemental Table S1.** Experimentally validated effector–host protein associations observed in coIP/MS data.

**Supplemental Table S2.** Biological processes targeted by *P. infestans* effectors.

**Supplemental Table S3.** N-terminally GFP-tagged PexRD12/31 effectors coimmunoprecipitate with a largely overlapping set of *N. benthamiana* vesicle trafficking proteins compared to FLAG-tagged effectors.

**Supplemental Table S4.** Primers used in this study.

**Supplemental Data Set S1.** A, *Nicotiana benthamiana proteins* identified by CoIP/MS screen of *P. infestans* RXLR effectors. B, Amino acid sequences of *N. benthamiana* proteins identified in coIP/MS screen of *P. infestans* RXLR effectors.

**Supplemental Data Set S2.** A, Consolidated coIP/MS data. B, GO term assignment of *N. benthamiana* proteins identified in coIP/MS screen.

**Supplemental Data Set S3.** *Nicotiana benthamiana* proteins in selected GO Biological Processes identified in coIP/MS of *P. infestans* RXLR effector families as presented in Supplemental Figure 3.

**Supplemental Data Set S4.** Anti-GFP coIP/MS data.

**Supplemental Data Set S5.** Coding nucleotide sequences of the fusion proteins used in this study.

**Supplemental Movie S1.** PexRD31 labels mobile cytosolic bodies in leaf cells.

**Supplemental Movie S2.** PexRD31-positive cytosolic puncta accumulate at haustoria.

**Supplemental Movie S3.** FYVE-labeled endosomes aggregate in leaf tissues colonized by *P. infestans.*

**Supplemental File S1**. RD12-31 family phylogenetic tree in Newick format.

**Supplemental File S2**. NbVAMP phylogenetic tree in Newick format.

## Supplementary Material

koab069_Supplementary_Data

## Notes

B.P., M.P.C., J.H.V., S.R., J.W., and S.K. involved in the conception, design, and writing the manuscript. B.P., M.P.C., J.W., T.O.B., M.H.S., J.S., S.S., A.A-.E.-H., R.C.-G., R.L.-D., Y.F.D., F.L.H.M., and A.M.E.J. responsible for data acquisition, analysis, and interpretation. B.P., M.P.C., T.O.B., R.C.-G., J.H.V., S.R., J.W., and S.K. revising and critically editing the manuscript.

The authors responsible for the distribution of materials integral to the findings presented in this article in accordance with the policy described in the Instructions for Authors (https://academic.oup.com/plcell) are: Sophien Kamoun (sophien.kamoun@tsl.ac.uk) and Joe Win (joe.win@tsl.ac.uk).
