# Supplementary material for: Host-interactor screens of Phytophthora infestans RXLR proteins reveal vesicle trafficking as a major effector-targeted process
Source: Plant Cell. 2021 Mar 2;33(5):1447–71. doi: 10.1093/plcell/koab069 (PMC8254500; doi:10.1093/plcell/koab069)
Supplement: koab069_Supplementary_Data [file koab069_supplementary_data.zip › tpc.00804.2020-s11.docx]

# **Supplemental Movie S1. PexRD31 labels mobile cytosolic bodies in leaf cells.** Confocal microscopy movie of a leaf epidermal cell of *Nicotiana benthamiana* expressing mCherry-PexRD31. The movie shows the mCherry channel. The first image of the movie is shown in Figure 6A. The 25 s movie was acquired over a *circa* 6 minute period, and consists of 171 sequential images displayed at a rate of *circa* 7 images per second. Each image shows a single optical section of 0.8 µm. Magnification and focus were adjusted throughout the movie to show different areas of a cell (from cell surface to cell central section), where mCherry signal labelled mobile cytosolic puncta, as well as cell and nuclear periphery. Live-cell imaging was performed with a laser-scanning confocal microscope three days after agroinfiltration. This supplemental movie supports Figure 6.

# **Supplemental Movie S2. PexRD31-positive cytosolic puncta accumulate at haustoria.** Confocal microscopy z-stack movie of a leaf epidermal cell of *N. benthamiana* expressing mCherry-PexRD31 in leaves colonized by *P. infestans* isolate 88069. The movie shows an overlay of the mCherry (magenta), chloroplast autofluorescence (blue), and bright field channels. The movie corresponds to the images shown in Figure 7A. The 3 s movie was acquired over a *circa* 1 minute period, and consists of 10 sequential images displayed at a rate of *circa* 4 images per second. Each image shows a single optical section of 0.8 µm. The z position was adjusted throughout the movie to show different areas of a cell, where mCherry-labelled cytosolic puncta, an mCherry-labelled haustorium, and unlabelled *P. infestans* hyphae are visible. Live-cell imaging was performed with a laser-scanning confocal microscope three days after infection. This supplemental movie supports Figure 7.

# **Supplemental Movie S3. FYVE-labelled endosomes aggregate in leaf tissues colonized by *Phytophthora infestans*.** Confocal microscopy movie of leaf epidermal cells of transgenic *N. benthamiana* expressing a 2xFYVE-GFP fusion (marker of PI3P-positive endosomes; cyan) in leaves colonized by transgenic *P. infestans* isolate 88069td (expressing a cytosolic tandem dimer red fluorescent protein [TdTomato]; magenta). The movie shows an overlay of the GFP (cyan), RFP (magenta), and chloroplast autofluorescence (blue) channels. The first image of the movie is shown in Figure 9B. The 11 s movie was acquired over a *circa* 18 minute period, and consists of 43 sequential images displayed at a rate of *circa* 4 images per second. Each image shows a maximal projection of 10 optical sections of 0.8 µm (z-stack of 8 µm). Global movement in the movie is due to sample drifting in the water between the glass slide and coverslip during imaging. Live-cell imaging was performed with a laser-scanning confocal microscope three days after infection. This supplemental movie supports Figure 9.
