# Supplementary material for: Host-interactor screens of Phytophthora infestans RXLR proteins reveal vesicle trafficking as a major effector-targeted process
Source: Plant Cell. 2021 Mar 2;33(5):1447–71. doi: 10.1093/plcell/koab069 (PMC8254500; doi:10.1093/plcell/koab069)
Supplement: koab069_Supplementary_Data [file koab069_supplementary_data.zip › tpc.00804.2020-s12.pdf]

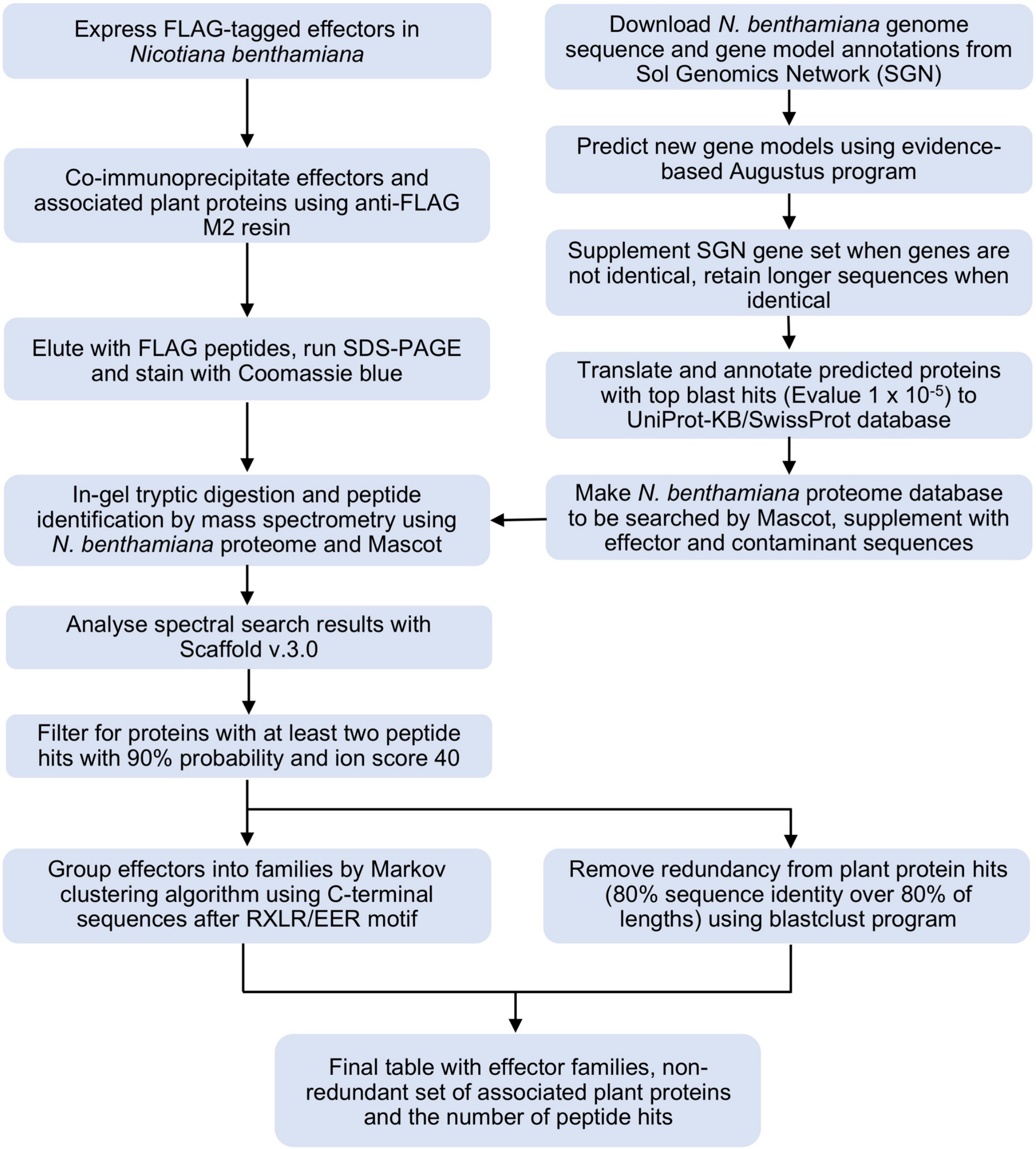

Supplemental Figure S1. Strategy to screen for effector-associated plant proteins in *Nicotiana benthamiana*. This supplemental figure supports Figures 1 and 2 in the main manuscript, showing how MS data used in the figures were obtained and analyzed.

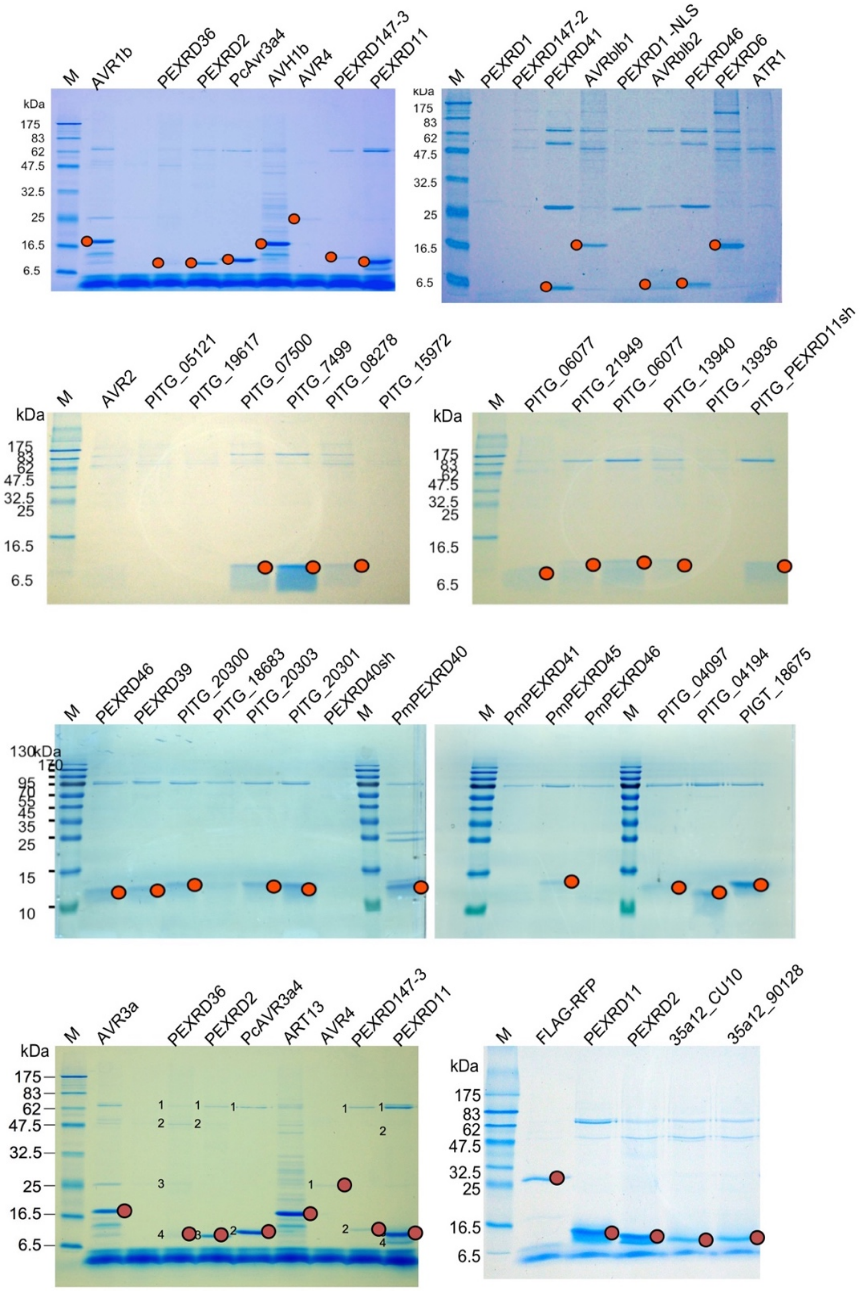

Supplemental Figure S2. Anti-FLAG immunoprecipitation efficiently purifies the effector fusions. Protein mixtures isolated by anti-FLAG immunoprecipitation (IP) were reduced and denatured in a Laemmli buffer, then subjected to SDS-PAGE and Coomassie blue staining. Approximate location of the band signals matching the expected size of the FLAG-tagged effector fusion are indicated with red filled circles. M = PageRuler standard protein molecular weight marker (Thermo Scientific) or Precision protein standards ladder (Bio-Rad). Numbers on the left side of the gels indicate the size of marker bands in kilodalton. This supplemental figure supports Figures 1 and 2 in the main manuscript providing additional evidence that intact FLAG-effector fusion proteins were recovered after the IP.

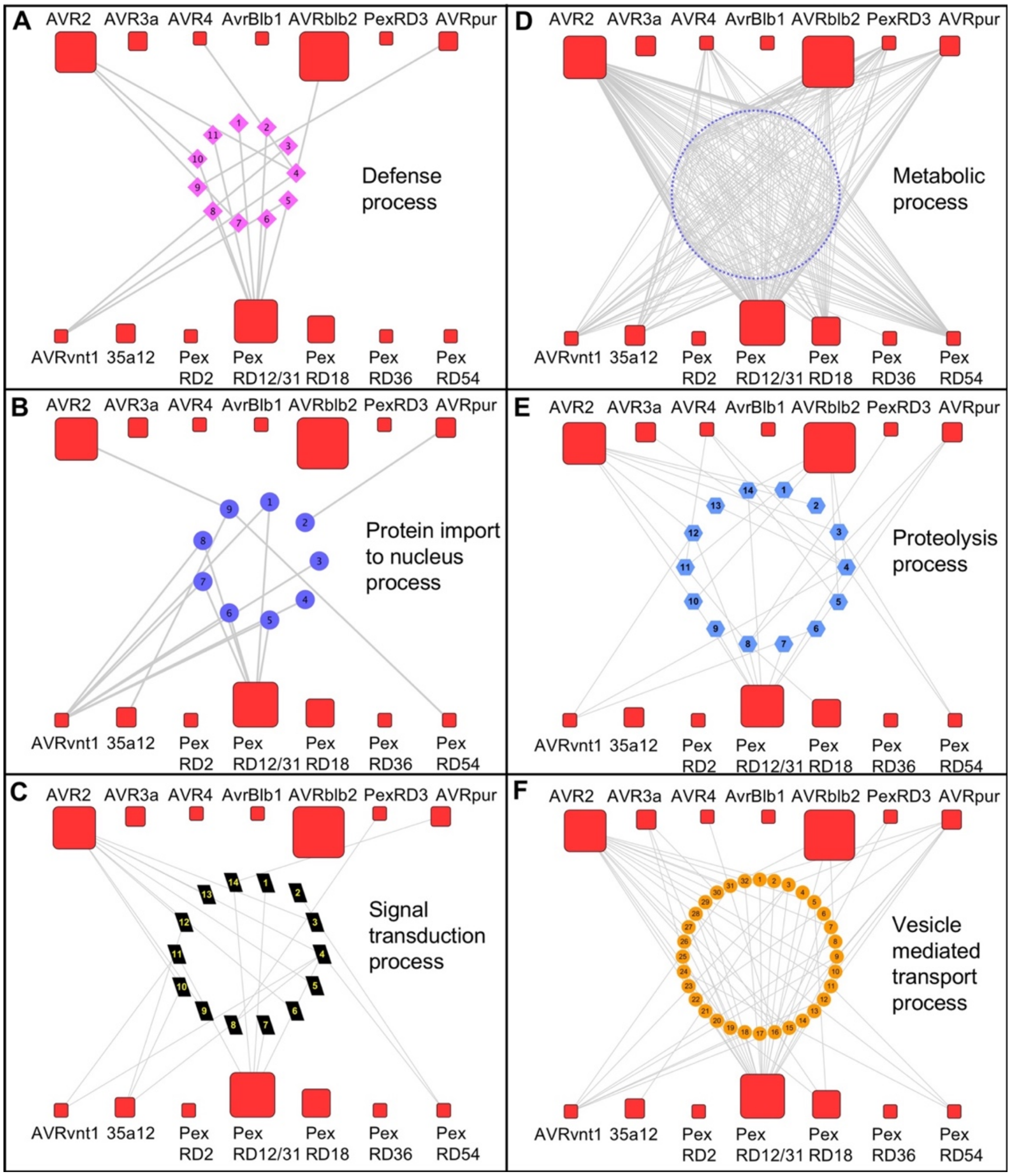

Supplemental Figure S3. Subnetworks of the RXLR effector interactome organized by GO terms. Subnetworks were shown with host proteins involved in (**A**) defense processes, (**B**) protein import to nucleus processes, (**C**) signal transduction processes, (**D**) metabolic processes, (**E**) proteolysis processes, (**F**) vesicle-mediated transport processes. Effector families are depicted in rounded red squares and the sizes of the squares correspond to the sizes of the effector families. This supplemental figure supports Figure 2 in the main manuscript providing extra subnetworks of selected biological processes. The identities of the proteins involved in these subnetworks is provided in Supplemental Data Set S3.

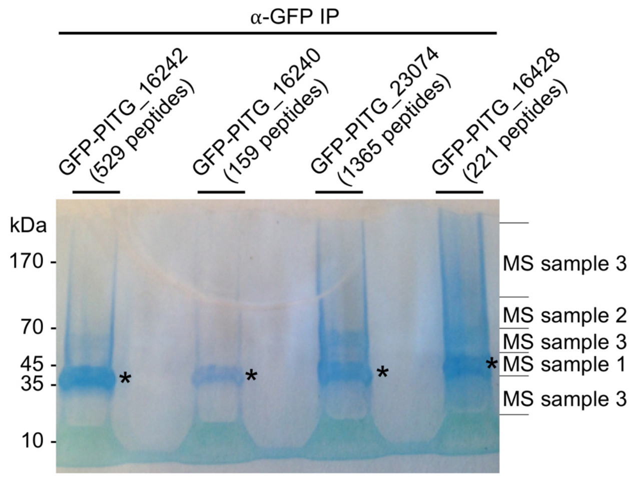

Supplemental Figure S4. Anti-GFP immunoprecipitation efficiently purifies GFP-tagged effector fusion proteins transiently expressed in *Nicotiana benthamiana* leaves. Protein mixtures isolated by anti-GFP immunoprecipitation were reduced and denatured in Laemmli buffer, then subjected to SDS-PAGE and Coomassie blue staining. For each GFP fusion, the number of peptides identified by LC-MS/MS and matching their sequence is indicated in parenthesis under the fusion protein names. The approximate sizes of selected proteins in PageRuler standard protein markers (Thermo Scientific) are indicated on the left in kilodalton (kDa). Black asterisks indicate the band signal matching the expected size of the GFP fusion proteins. For each lane, the area of gel cut and processed as an independent sample for LC-MS/MS is indicated on the right (numbered MS sample 1 to 3). This supplemental figure supports Supplemental Data Set S4 and Figure 5 in the main manuscript confirming that the fluorescent fusion proteins are intact in *N. benthamiana* leaf cells.

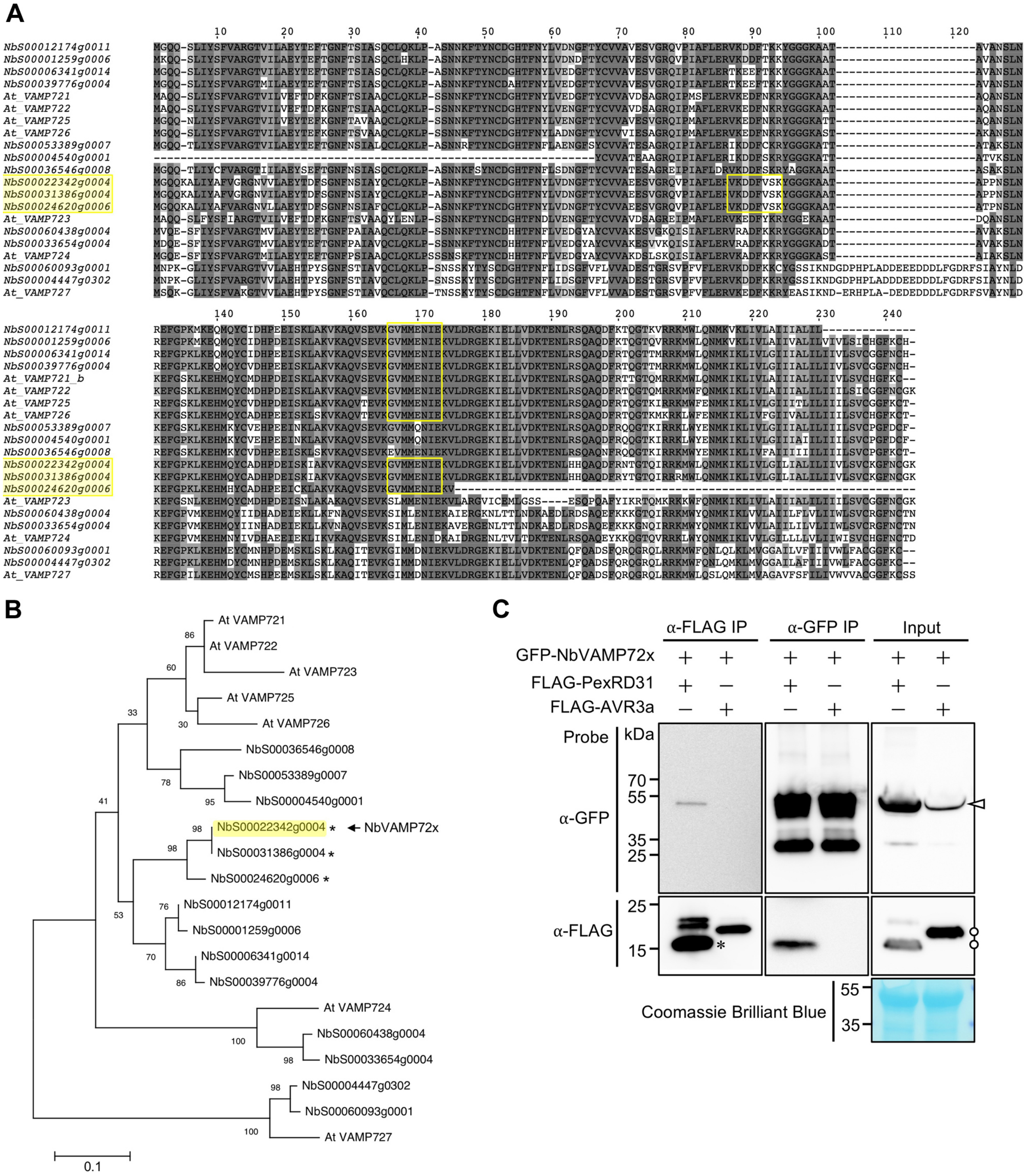

Supplemental Figure S5. NbVAMPs associated with PexRD12/31 effectors belong to the VAMP72 family in *Nicotiana benthamiana.* (A) Multiple sequence alignment of VAMP 72 family from *N. benthamiana* (SolGenomics Network database accession numbers were shown) and *Arabidopsis thaliana.* GenBank accession numbers of *A. thaliana* VAMPs (in parenthesis): At_VAMP721_b (NP_171967.1), At_VAMP722 (P47192.2), At_VAMP723 (NP_850201.1), At_VAMP724 (O23429.2), At_VAMP725 (O48850.2), At_VAMP726 (Q9MAS5.2), At_VAMP727 (NP_001078283.1). *N. benthamiana* VAMPs identified by two peptide hits in MS spectral searches are highlighted in yellow. Peptide hits were shown in yellow rectangle box.

(B) Phylogenetic tree of VAMP72 family from *N. benthamiana* and *A. thaliana*. The phylogeny was inferred using the Maximum Likelihood method and JTT matrix-based model. The tree with the highest log likelihood (-930.02) is shown. The percentage of trees in which the associated taxa clustered together is shown next to the branches. The tree is drawn to scale, with branch lengths measured in the number of substitutions per site. This analysis involved 21 amino acid sequences. All positions containing gaps and missing data were eliminated (complete deletion option). There was a total of 88 positions in the final dataset. Cloned NbVAMP is highlighted in yellow; * = VAMP identified by two peptide hits. The Newick tree file is provided in Supplemental File S2.

(C) Immunoblots show FLAG-tagged PexRD31 effector fusion protein coimmunoprecipitates with GFP-tagged NbVAMP72x transiently co-expressed in *N. benthamiana*. Approximate molecular weights of the proteins are shown on the left in kDa. Open arrowhead shows the expected size of the GFP-NbVAMP72x bands and open circles show the expected sizes of the FLAG-tagged effector bands. IP = immunoprecipitation. This supplemental figure supports Figure 4 in the main manuscript by describing how a specific NbVAMP was identified as an interactor of PexRD12/31 family, and by showing that FLAG-PexRD31 (indicated with *) can be detected after coIP in this experiment. PexRD31 (PITG_23074 in Figure 4) was not detectable in anti-FLAG coIP in the immunoblot used in Figure 4.

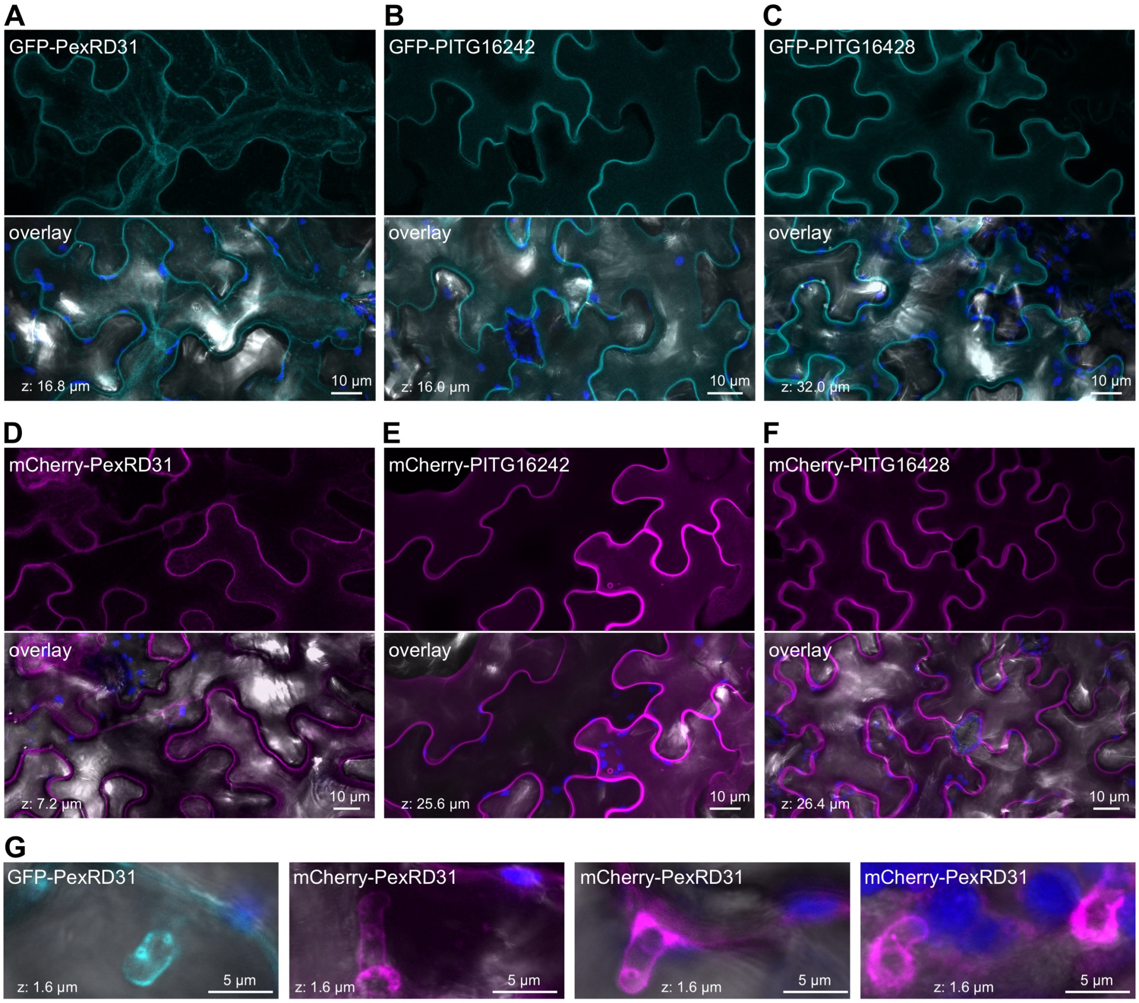

Supplemental Figure S6. PexRD12/31 effectors accumulate mainly at the cell periphery and around haustoria during *Phytophthora infestans* infection. Live-cell imaging of **(A)** green fluorescent protein (GFP)-PexRD31 **(B)** GFP-PITG16242, **(C)** GFP-PITG16428, **(D)** mCherry-PexRD31, **(E)** mCherry-PITG16242, **(F)** mCherry-PITG16428 fusion proteins transiently expressed in *Nicotiana benthamiana* leaves, and **(G)** confocal microscopy images of *P. infestans* infected *N. benthamiana* leaf epidermal cells expressing GFP-PexRD31 (left-most panel) and mCherry-PexRD31. The overlay images show a close-up of PexRD31-positive bodies associated with haustoria (finger-like projections). Live-cell imaging was performed with a laser-scanning confocal microscope three days after infiltration. GFP and chlorophyll were excited at 488 nm; mCherry was excited at 561 nm. GFP (cyan), mCherry (magenta), and chlorophyll (blue) fluorescence were collected between 505 and 525 nm, 580 and 620 nm, and 680 and 700 nm, respectively. Images are maximal projections of up to 40 optical sections (maximal z-stack of 32.0 µm). The overlay panels combine fluorescent protein (GFP or mCherry), chlorophyll, and bright field images. (A)-(F) support Figure 5 in the main manuscript showing the accumulation of fluorescence from individually expressed effector proteins with different fluorescence tags and (G) supports Figure 7 providing additional images shown in Figure 7A.

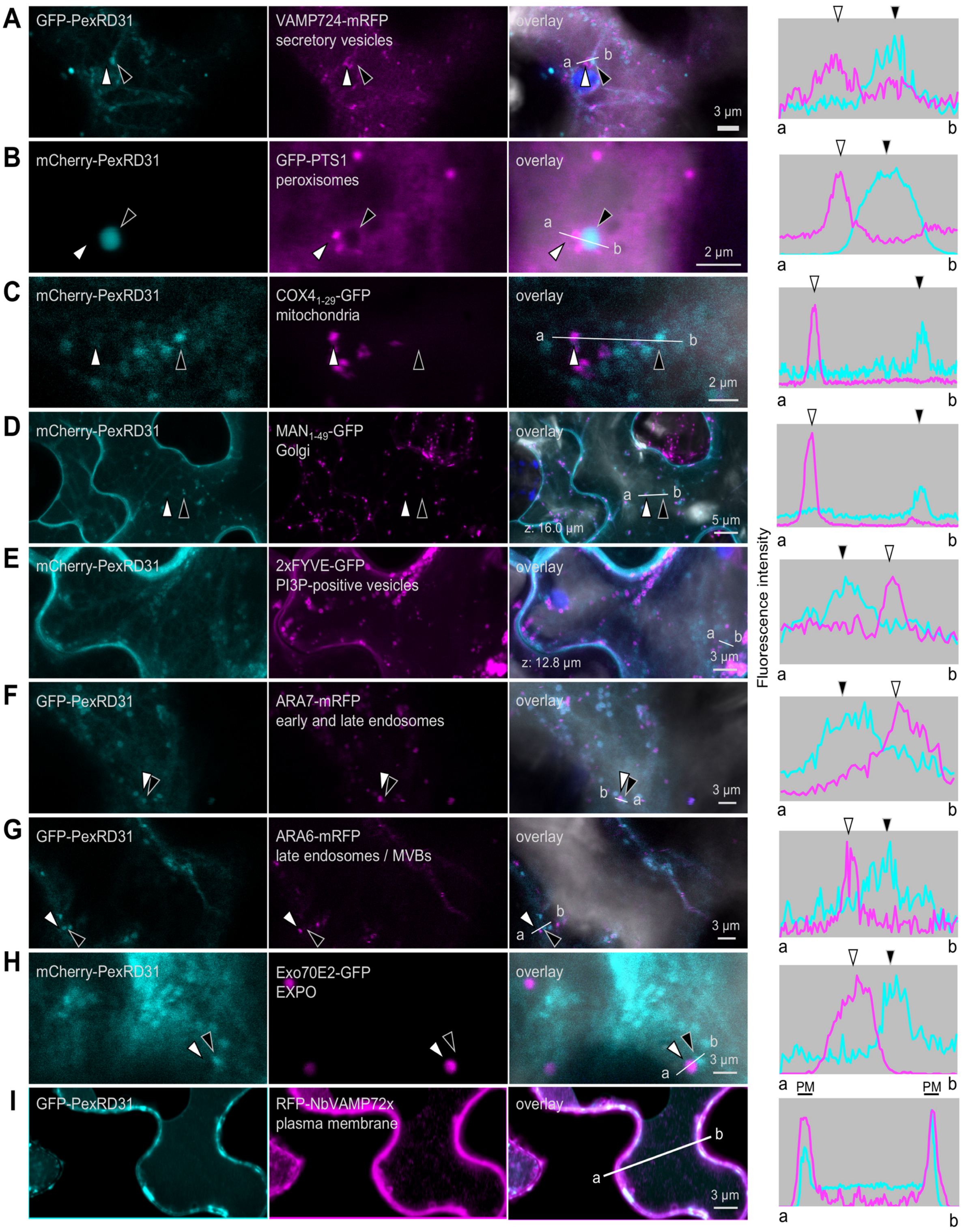

Supplemental Figure S7. PexRD31 does not accumulate in the main cell compartments but co-accumulate with NbVAMP72x at the plasma membrane. Live-cell imaging of **(A)** green fluorescent protein (GFP)-PexRD31 and VAMP724-monomeric red fluorescent protein (mRFP) (secretory vesicle marker), **(B)** mCherry-PexRD31 and GFP-PTS1 (peroxisome marker), **(C)** mCherry-PexRD31 and COX41-29-GFP

(mitochondrion marker), **(D)** mCherry-PexRD31 and MAN11-49-GFP (Golgi marker), **(E)** mCherry-PexRD31and 2xFYVE-GFP (Phosphatidylinositol-3-Phosphate [PI3P]-positive vesicle marker), **(F)** GFP-PexRD31 and mRFP-ARA7 (endosome marker), **(G)** GFP-PexRD31 and ARA6-mRFP (endosome and multivesicular body marker), **(H)** mCherry-PexRD31 and EXO70E2-GFP (Exocyst-positive organelle [EXPO] marker), and **(I)** GFP-PexRD31 and RFP-NbVAMP72x co-accumulating at the plasma membrane. All experiments were performed in *Nicotiana benthamiana* leaves. Proteins were expressed in leaf cells by agroinfiltration. Live-cell imaging was performed with a laser-scanning confocal microscope three days after infiltration. GFP and chlorophyll were excited at 488 nm; mCherry was excited at 561 nm. GFP, mCherry, and chlorophyll fluorescence were collected between 505 and 525 nm, 580 and 620 nm, and 680 and 700 nm, respectively. Images are single optical sections of 0.8 µm or maximal projections of up to 20 optical sections (max. z-stack: 16.0 µm). The overlay panel combines GFP, mCherry, chlorophyll, and bright field channels. The right-hand side panel show relative fluorescence intensity plots of the GFP and the mCherry along the line from a to b depicted in the corresponding overlay panel. In the intensity plots, white and black arrowheads indicate fluorescence peaks as visible in the overlay panel. Note that we adapted the magnification to the nature (size, mobility, or number) of the cellular compartments that is illustrated. This supplemental figure supports Figures 4 and 5 in the main manuscript, showing that both PexRD31 and NbVAMP72x accumulate at the plasma membrane.

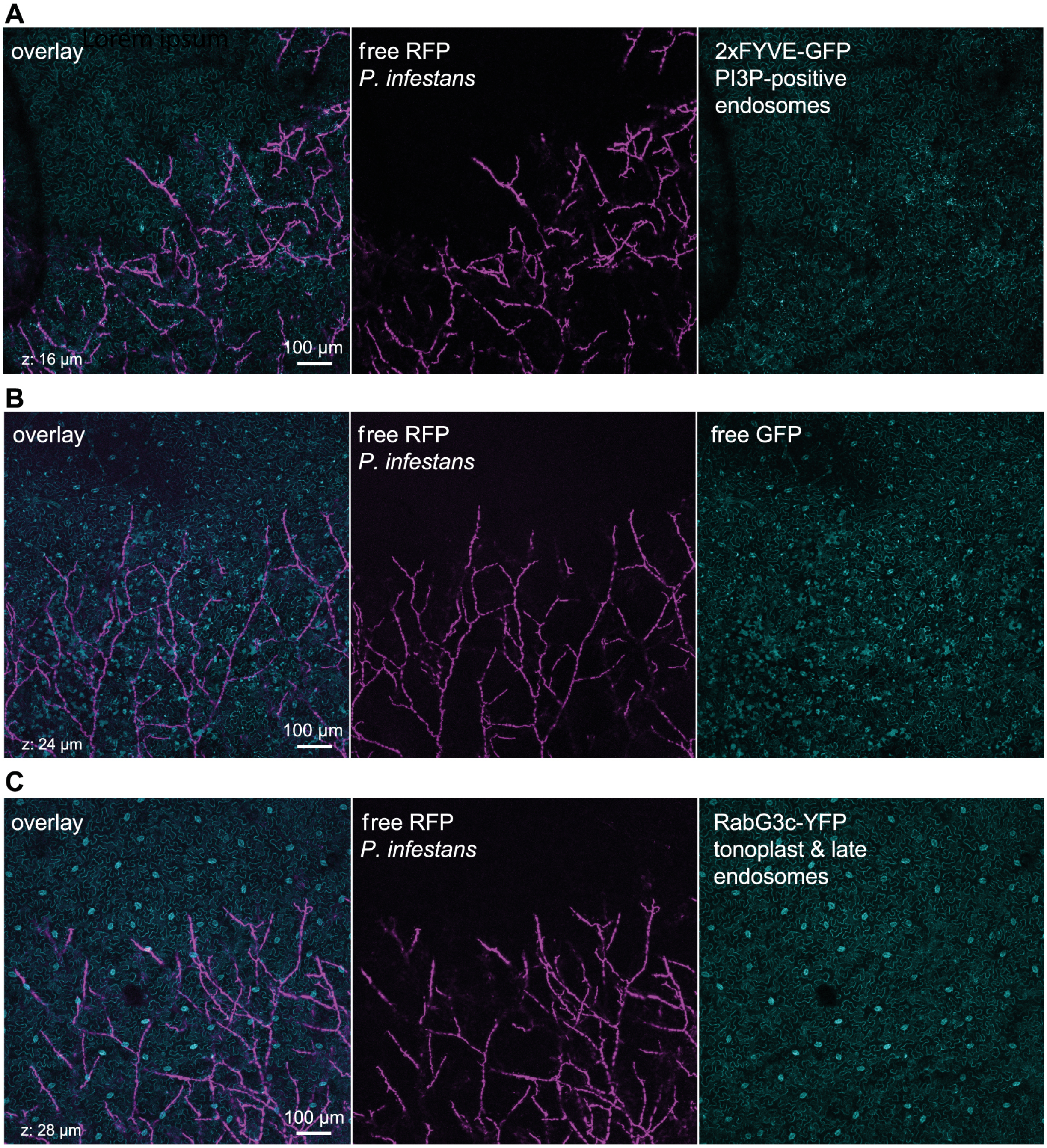

Supplemental Figure S8. *Phytophthora infestans* biotrophic colonization does not trigger the formation of punctate signals in the nucleocytosolic, late endosomal, and tonoplast compartments. Live cell imaging of **(A)** a 2xFYVE-GFP fusion (marker of PI3P-positive endosomes), **(B)** a free GFP (marker of the nucleus and cytosol), and **(C)** a YFP-RabG3c fusion (marker of late endosomes and tonoplast) in *N. benthamiana* leaf cells colonized by *P. infestans* isolate 88069td. Leaves of stable transgenic *N. benthamiana* plants were drop inoculated by zoospores of *P. infestans* isolate 88069td. Live-cell imaging was performed with a laser-scanning confocal microscope three days after inoculation. GFP, YFP, and RFP were excited at 488 nm, 514 nm, and 561 nm, respectively. GFP (cyan), YFP (cyan), and RFP (magenta) fluorescence were collected

between 505 and 525 nm, 525 and 550 nm, and 580 and 620 nm, respectively. Images are maximal projections of up to 35 optical sections (max. z-stack of 28 μm). The overlay panels on the left-hand side combines either **(A-B)** the GFP and RFP channels or **(C)** the YFP and RFP channels. Note the presence of GFP puncta in **(A)** (positive control) but not in **(B-C)**. This supplemental figure supports Figure 9 in the main manuscript describing that the FYVE-labelled punctate formation affected by *P. infestans* infection was not due to changes in nucleocytosolic, late endosomal, and tonoplast compartments that are labelled by YFP-RabG3c.

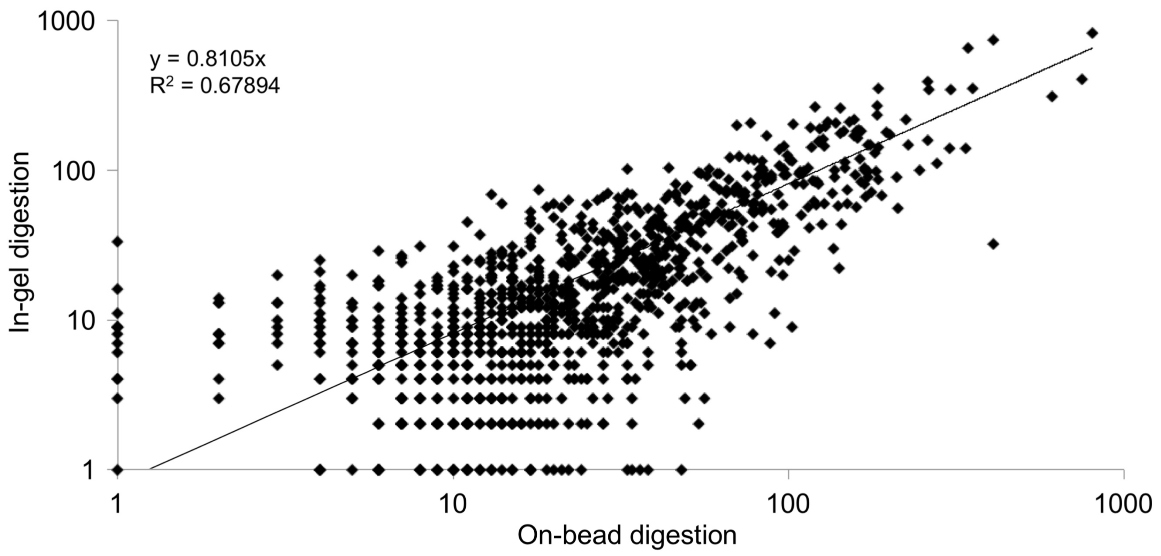

Supplemental Figure S9. In-gel and on-bead trypsin digestion methods yield similar data. Total MS spectrum count values (all samples merged, added of one unit) from in-gel and on-beads trypsin digestion methods were plotted using Microsoft Excel 'Marked Scatter' graph function. Linear regression and associated parameters were calculated using the same software. Both axes have a Log scale. This supplemental figure supports Supplemental Data Set S4 providing a graphical evidence that two methods of trypsin digestion result in similar data.

Supplemental Table S1. Experimentally validated effector-host protein associations observed in coIP/MS data.

Effector	Plant protein	Number of associated effector families	References
**AVRblb2**	Cysteine proteinase RD21a (C14)	3	Bozkurt et al., 2011
**AVR2**	BSL1 serine/threonine protein phosphatase	1	Saunders et al., 2012
**AVR2**	BSL3 serine/threonine-protein phosphatase	1	Turnbull et al., 2019
**AVR3a**	Dynamin 2B	5	Chaparro-Garcia et al., 2015
**PexRD54**	ATG8	N/A	Maqbool et al., 2016
**PexRD54**	Ras-related protein RabE1c	4	Pandey et al., 2020
**PexRD12/31 (7 effectors)**	Vesicle associated membrane protein 7B (NbVAMP72x)	2	This study

Supplemental Table S2. Biological processes targeted by *Phytophthora infestans* effectors

Biological process[1]	Effector family[2]													
	AVR2	AVR3a	AVR4	AVRblb1	AVRblb2	PexRD3	AVRpur	AVRvnt1	PexRD12	PexRD18	PexRD2	PexRD36	PexRD54	35a12
ATP hydrolysis coupled proton transport	3		1		2		1	4	5	1		2		
ATP synthesis coupled proton transport	7	1	5		5		2	1	4	2				1
Actin cytoskeleton organization					1	1	2	2	2	1				
Apoptotic process									1					
Cell development	2		1		1	1	1	1	1					
Cell redox homeostasis	4													
Cell wall biogenesis	1				1	1	1	1	5	1				
Cell wall modification	2													
Cellular response to reactive oxygen Species	1													
Chloroplast organization	1								1					
Cytokinesis					1				3					
DNA repair						1	1		2					
Defense response	3		1		1		1	3	7					
Detection of biotic stimulus	1									1				
Electron transport	2								1					
Metabolic process	67		15		2	23	23	19	66	2		1		16
Methylation	1													
Microtubule cytoskeleton organization	2		1		1		2	2	3	1				
Microtubule-based movement	2		1		1		2	3	3					1
Negative regulation of peptidase activity	2				2	1		1						
Nucleosome assembly	3	2	1		3		1		1	1				
Oxidation-reduction process	3				1				1					
Phagocytosis	1													
Photosynthesis	27		7	1	12	4	4	7	12	8	1	2		8
Proteasome-mediated ubiquitin-dependent protein catabolic process	13		2		2	2	14	11	28	2				1
Protein folding	25	4	6	4	1	3	14	1	2	7	4	2		7
Protein Import into chloroplast stroma	1					1			2					
Protein import into nucleus	1						1	7	5					1
Protein phosphorylation									1					
Protein stabilization									1					
Protein targeting to chloroplast	1						1		1					
Protein targeting to mitochondrion	1													
Protein targeting to vacuole	1													
Proteolysis	8	1	3		5	1	3	3	8	1				
RNA processing	5	1	2		2	1	2	2	5	3				
RNA transcription					2									
RNA-dependent DNA replication	1					1	1		1					
Regulation of cellular process			1		1									
Removal of superoxide radicals	1													
Response to heat							1							
Response to stress	1													
Signal transduction	7					1	1	2	5					2
Thylakoid membrane organization			1		1				3					
Translation	51		4		49	3	6	67	82	14	2	1		11
Transport	1		4		6	5	3	8	26	3	1			
Unassigned	19		2		3	8	4	4	18	4				
Vesicle-mediated transport	14	3	1		3	2	7	7	24	3				1

[1] Biological processes were annotated according to terms described by Gene Ontology Consortium.
[2] The numbers represent number of plant proteins in a biological process that are associated with each effector family.

Supplemental Table S3. N-terminally GFP-tagged PexRD12/31 effectors co-immunoprecipitate with a largely overlapping set of *Nicotiana benthamiana* vesicle trafficking proteins compared to FLAG-tagged effectors

N. benthamiana protein annotation	Effectors used[1]																		Sequence identifier
	Anti-GFP IPs						Anti-FLAG IPs												
	GFP-PITG_16245	GFP-PITG_16242	GFP-PITG_23074	GFP-PITG_16428	Total no. peptides	Other GFP fusions[2]	FLAG-PITG_16245	FLAG-PITG_16233	FLAG-PITG_16235	FLAG-PITG 16242	FLAG-PITG_16409	FLAG-PITG_16243	FLAG-PITG 23069	FLAG-PITG_23074	FLAG-PITG_16428	Total no. peptides	Other FLAG fusions[3]	No. effector families associated[4]	
Vesicle fusing ATPase[5]			1	4	5				5	4	3	1	2	2	10	27	4	2	NbS00011575g0012
Transmembrane emp24 domain-containing protein A[5]				6	6						2					2		1	NICBE_138070
Dynamin 2B		2	4	1	7	16	7			1						8	7	5	NICBE_074039
Syntaxin[5]			13	6	19										5	5	2	1	NbS00027157g0003
ADP-ribosylation factor 2[5]	9		6	4	21	12									2	2	11	2	NICBE_020747
Vacuolar-sorting receptor 3[5]			9	13	22				1	2					2	5		1	NICBE_083936
Putative phagocytic receptor 1b[5]		1	13	9	23										1	1	9	3	NICBE_351046
Ras-related protein RABA1f[5]		1	9	16	26										4	4	4	2	NICBE_128666
Vesicle associated membrane protein 7B[5]		5	2	19	26					1					2	3	4	2	NbS00022342g0004
Extended synaptotagmin-3[5]			9	19	28										1	1	2	1	NICBE_418648
Exocyst complex component 5[5]		15	21	2	38		4									4		1	NICBE_108112
Sec7 guanine nucleotide exchange factor[5]		7	21	12	40										2	2	2	2	NbS00049277g0005
Ras related protein Rab 2 A[5]		6	34	5	45				1	1			1		2	5	9	3	NbS00004361g0010
Coatomer subunit delta[5]	1	9	18	25	53								2		1	3	4	1	NICBE_222296
Coatomer alpha subunit protein	7	9	19	27	62		5								3	8	4	5	NbS00003584g0003
Exocyst complex component 3[5]	1	5	29	35	70		2									2		1	NICBE_298622
Probable exocyst complex component 4[5]		11	39	43	93		2						1	1		4	2	1	NICBE_251150
Exocyst complex component SEC3A[5]		14	46		105		2									2		1	NICBE_053349
Coatomer subunit alpha-1[5]	15	33	49	36	133	23	7								3	10	9	4	NICBE_286236
Coatomer subunit beta-2[5]	16	24	43	53	136	21	1								1	2	11	4	NICBE_204349
Coatomer subunit gamma[5]	3	23	71	72	169	16	13	5	1				2		7	28	7	3	NbS00000812g00012
ARF guanine nucleotide exchange factor 2[5]		52	88	57	197		1									1	2	1	NbS00006288g0001
Pattern formation protein EMB30[5]	1	14<br>5	42	23	211		2							1	2	5		1	NICBE_346430
	12	40	92	16<br>4	308	14	8						1		2	11	7	5	NICBE_323561
SEC1 family transport protein SLY1[5]							3									3		1	NICBE_170643
SNAP25 homologous protein SNAP33													1		2	3	1	1	NICBE_352850
Annexin D1[5]						4	4						3			7	9	2	NICBE_369402
Dynamin related protein 1E[5]						13	1									1	6	2	NbS00056353g0008
Ras-related protein RABE1c[5] (Rab8a)						12			1	1	1	1	2	1	1	8	15	4	NICBE_214722
CASP protein							6	1							6	13	19	6	NICBE_048655

[1] Numbers shown in the table refer to the number of peptide hits against *N. benthamiana* protein identified by coIP/MS of effectors.
[2] Other anti-GFP coIP/MS datasets extracted from Petre *et al.*, 2015, 2016 (negative controls). In total, 36 experiments are included.
[3] Other anti-FLAG coIP/MS datasets from this study (negative controls).
[4] Number of associated effector families was acquired from Dataset 1 and 2.
[5] Plant protein that shows association with less than five effector families.

Supplemental Table S4. Primers used in this study

**Primer ID**	**Primer sequence (5' to 3')**
PITG16242F	CACCGAAGACACAATGTGGCTTAGTCGTGTTACAAATTG
PITG16242R	CTACGAAGACGGAAGCTTAGTGTTTGTTCCGCCATTCGG
PITG16428F	CACCGAAGACACAATGCTGATCAAAACCTTCGAGTC
PITG16428R	CTACGAAGACGGAAGCTTAGAAACGTTTTAGTCGGTTGTTGC
PITG23074F	CACCGAAGACACAATGATTTTTCGCCGATTTACGGATTG
PITG23074R	CTACGAAGACGGAAGCTTATAGTTTGTCCCGCCACTTTTTCG
PITG16245F	CACCGAAGACACAATGCTGCTTAATGGTATGACAG
PITG16245R	CTACGAAGACGGAAGCTTACTGGTTCTTCCACCACTTC
NbVAMP72x_F	CACCGGTCAGCAGAAGGCTTTGATCT
NbVAMP72x_R	TTACTTTCCACAATTGAATCCCT
