# Supplementary material for: Host-interactor screens of Phytophthora infestans RXLR proteins reveal vesicle trafficking as a major effector-targeted process
Source: Plant Cell. 2021 Mar 2;33(5):1447–71. doi: 10.1093/plcell/koab069 (PMC8254500; doi:10.1093/plcell/koab069)
Supplement: koab069_Supplementary_Data [file koab069_supplementary_data.zip › tpc.00804.2020-s14.pdf]

Host-interactor screens of *Phytophthora infestans* RXLR proteins reveal vesicle trafficking as a major effector-targeted process

Benjamin Petre, Mauricio P. Contreras, Tolga O. Bozkurt, Martin H. Schattat, Jan Sklenar, Sebastian Schornack, Ahmed Abd-El-Haliem, Roger Castells-Graells, Rosa Lozano-Duran, Yasin F. Dagdas, Frank L. H. Menke, Alexandra M. E. Jones, Jack H. Vossen, Silke Robatzek, Sophien Kamoun, Joe Win

Corresponding authors: Sophien Kamoun (sophien.kamoun@tsl.ac.uk) and Joe Win (joe.win@tsl.ac.uk).

Review timeline:

**TPC2020-LSB-00804**	Submission received:	Sept. 28, 2020
	1st Decision:	Nov. 8, 2020 *revision requested*
**TPC2020-LSB-00804R1**	1st Revision received:	Feb. 3, 2021
	2nd Decision:	Feb. 9, 2021 *acceptance pending, sent to science editor*
	Final acceptance:	Feb. 19, 2021

REPORT: (The report shows the major requests for revision and author responses. Minor comments for revision and miscellaneous correspondence are not included. The original format may not be reflected in this compilation, but the reviewer comments and author responses are not edited, except to correct minor typographical or spelling errors that could be a source of ambiguity.)

TPC2020-LSB-00804 1st Editorial decision – *revision requested* **Nov. 8, 2020**

We have received reviews of your manuscript entitled "Host-interactor screens of *Phytophthora infestans* RXLR proteins reveal vesicle trafficking as a major effector-targeted process." Thank you for submitting your best work to The Plant Cell. The editorial board agrees that the work you describe is substantive, falls within the scope of the journal, and may become acceptable for publication, pending revision and potential re-review.

We ask you to pay attention to the following points in preparing your revision:

1) All claims for co-localization of proteins need to be supported by quantitative analyses of the images.

2) Please use cyan and magenta instead of green and red for false color labelling to make overlay images legible for those with color vision deficiencies.

3) Note reviewer concerns about actual localization of the markers you used and adjust your conclusions accordingly. More generally, be more cautious about what can be concluded with confidence about your localizations.

4) Do not crop haustoria images as tightly so that the cellular context can be better understood.

5) The coIP of RD31 effectors with NbVAMP72x wasn't really substantiated, and is inconsistent with localization data. Do you have any confirmatory data for this interaction?

6) Provide a negative control for Figure 7, as requested by Reviewer #1.

-- Reviewer comments:

[Reviewer comments shown below along with author responses]

TPC2020-LSB-00804R1 1st Revision received **Feb. 3, 2021**

Reviewer comments and **author responses**:

Editor Comments:

Editor comment 1: All claims for co-localization of proteins need to be supported by quantitative analyses of the images.

Authors response: Done. We have added quantitative data for all colocalization analyses (see Figures 5, 6, 7, and Supplemental Figure 7) and we have modified the text accordingly by adding a section on quantification of colocalization data (see lines 906-918). We systematically used intensity plots to quantify the distribution of fluorescent signal over discrete cell compartments (all figures), and used the Fiji coloc2 plugin to automatically assess pixel intensity correlation over a full image or selected image areas (see Figures 5 and 7).

In addition, we have compiled and have made available all the raw data that pertain to microscopy analyses (raw images and quantitative analysis reports) in the Zenodo repository, that can be accessed here (https://doi.org/10.5281/zenodo.4497844). We mentioned this in the methods (see lines 937-939).

Editor comment 2: Please use cyan and magenta instead of green and red for false color labelling to make overlay images legible for those with color vision deficiencies.

Authors response: Done. We have redrawn all the figures that include microscopy images (see Figures 5 to 9 and Supplemental Figures 6 to 8), using cyan and magenta, as suggested. While redrawing these figures, we have also uncropped images to address Editor comment 4.

Editor comment 3: Note reviewer concerns about actual localization of the markers you used and adjust your conclusions accordingly. More generally, be more cautious about what can be concluded with confidence about your localizations.

Authors response: We have addressed these issues as follows (see also our response to Reviewer 2 comment 6).

We have modified the text to be more cautious about the compartments labelled by ARA6, ARA7, and RabC1. We no longer refer to RabC1-positive vesicles as components of the post-Golgi network, but simply refer to RabC1-positive bodies. Consequently, we have modified our conclusion regarding the localization of PexRD31, and now conclude that PexRD31 accumulates in RabC1-positive mobile bodies (see lines 402, 406, 411, 658-660 and Figure 6).

Editor comment 4: Do not crop haustoria images as tightly so that the cellular context can be better understood.

Authors response: Done. The manuscript now includes uncropped images. When high magnification was necessary, we included inserts with close-up images (see Figure 7). Note that all raw microscopy data is now available to readers (see our response to Editor comment 1).

Editor comment 5: The coIP of RD31 effectors with NbVAMP72x wasn't really substantiated, and is inconsistent with localization data. Do you have any confirmatory data for this interaction?

Authors response: Yes. We have now added a new section in the results (lines 414-426) and a confocal figure (Supplemental Figure 7I), showing that fluorescence signal from GFP-tagged PexRD31 overlaps with that from RFP-tagged NbVAMP72x at the plasma membrane. We conclude that PexRD12/31 effectors and NbVAMP72x accumulate in the same subcellular compartment in *N. benthamiana* leaf cells, providing an opportunity for their interaction. We have also included new coIP results that further substantiate the physical association observed between PexRD31 and NbVAMP72x (see Supplemental Figure 5C, see also our response to Reviewer 1 comment 1).

Editor comment 6: Provide a negative control for Figure 7, as requested by Reviewer #1.

Authors response: Done. We have included a control condition with a cytosolic fluorescent protein in the revised manuscript (see Figure 7F and lines 449-451; see also our response to Reviewer 1 comment 2)

Editor comment 7 (journal requirements):The Plant Cell now requires authors to complete and submit an author revisions checklist upon submission of a revised manuscript. The aim of the checklist is to aid authors in preparing a high quality manuscript, facilitate the review and assessment of revised manuscripts, and help to ensure that journal standards are maintained across the board. If your manuscript is accepted, the completed checklist will be published as supplemental material attached to the article online. Please download a copy of the checklist (pdf fillable form) at this link, for submission with your revised manuscript: https://tpc.msubmit.net/html/Author_Revisions_Checklist.pdf.

Authors response: Done. We have completed the checklist and included it with this resubmission.

Editor comment 8 (journal requirements): Supplemental materials should be restricted to large datasets and tables, presentation of replicates, and validation of reagents, methods, or genotypes. Any data that are used to support the major claims must be in the main manuscript. Supplemental figure legends must indicate what figure in the main manuscript is supported by the supplemental data presented. Please justify how each of the supplemental figures meet the criteria.

Authors response: We have carefully considered the inclusion of supplemental materials and we hope that the journal requirements are met. We also added extra sentences in the figure legends describing which main figure is supported by the supplemental figure and justification for their inclusion.

Editor comment 9 (journal requirements): Sampling methods and nature of "biological replicates" should be described precisely (i.e. different plants, parts of plants, pooled tissue, independent pools of tissue, sampled at different times, etc), along with a clear description of and rationale for any statistical analyses conducted. The reader should know exactly what was sampled; what forms the basis of the calculation of any means and statistical parameters reported. This is also necessary to ensure that proper statistical analysis was conducted.

Author response: We have explicitly mentioned our sampling methods throughout the main text, figure legends and in method sections. We have added statistical methods we used in any quantification of data reported in the main text (e.g., lines 880-882, 886-888, 915-918) and the figure legends.

Reviewer #1:

Reviewer 1 comment 1 (key point): Please discuss the finding that although the RD31 effectors coIP with NbVAMP72x, they do not colocalise with it, nor indeed does RabC1.

Authors response: Although we do not have the evidence of PexRD31 and NbVAMP72x colocalizing in endocytic vesicles, we previously obtained some results that showed that PexRD31 and NbVAMP72x co-accumulate at the plasma membrane. However, we did not formalize the images to include them in the first version of the manuscript. The revised manuscript now includes these results (see supplemental figure 7I), as well as new coIP data that support the association between PexRD31 and NbVAMP72x (see supplemental Figure 5C). See also our response to Editor comment 5.

Reviewer 1 comment 2 (key point)

Comment 2.1: The authors need to provide a negative control (ideally another effector fusion) for the perihaustorial localisation of the effectors in Fig7 in order to make any point about whether this localisation is a specific property of these effectors.

Comment 2.2: In lines 553-557 it is stated that "Some of these puncta were immobile and were in proximity to the haustorial interface, enabling us to image them at higher resolution. This revealed that mCherry-PexRD31 produced a sharp circular fluorescent signal that can be observed near haustoria". There is only a single one in Fig7 so to demonstrate this was not just random chance the authors should provide more images with the punctae visible at haustoria, in supplementary data or a linked online repository.

Author response

Comment 2.1: Done. This point has now been addressed by adding a negative control in Figure 7 (see our response to Editor comment 6).

Comment 2.2: We have assembled additional images that show haustoria associated with PexRD31 bodies from four independent microscopy assays (performed at four different dates: 28/08/2015, 05/02/2016, 07/03/2016, and 08/03/2016, see Supplemental Figure 6G) and included them along with the raw data on Zenodo repository (see also our response to Editor comment 1). These images come in addition to the one shown in Figure 7, which also show haustoria associated with PexRD31 bodies, and which come from other independent microscopy assays (02/07/2015 and 16/02/2016). We have also included a z-stack movie that better shows how these bodies distribute around haustoria dimensionally (see Supplemental Movie 2 and line 442).

Reviewer 1 comment 3 (key point): Is there a logical evolutionary basis for effectors of the same family targeting quite different and sequence-unrelated proteins that happen to be involved in a common pathway? The authors should discuss this since this commonality is one of the main points of the work.

Authors response: Thank you for bringing up this point. We have discussed it in the discussion (line 596- 610). One could imagine that different members of one effector family could evolve to target different protein components involved in a common pathway. This would create redundancies in effector perturbation of an important pathway, enabling the pathogen to adopt a more robust infection strategy.

Reviewer 1 comment 4 (minor point): Line 104: put 'for example' at the front of the list of references for effector characteriation since the list is not exhaustive.

Authors response: We modified the text accordingly (see line 108).

Reviewer 1 comment 5 (minor point): Line 128: Wang et al 2018 list more than four effectors accumulating around haustoria.

Authors response: We have modified the text accordingly (see line 132).

Reviewer 1 comment 6 (minor point): Line 139: 'to identify' better than 'at identifying'

Authors response: Modified as suggested (See line 142).

Reviewer 1 comment 7 (minor point): Line 168-170: Please provide more detail on the effector selection criteria. It is stated that the set was enriched for those induced during infection, what were the reasons for choosing the ones that weren't?

Authors response: As we stated in the text (lines 170-172), we selected RXLR effector families based on (1) being induced during infection and (2) having AVR activities. We have made the text clearer (lines 170-172). Some effectors in AVR families may not be upregulated during infection above steady-state levels but were still selected for our screen.

Reviewer 1 comment 8 (minor point)

Comment 8.1: Fig S6: The punctae clearly visible in the FP-PexRD31 Fig6A and the left column of FigS7 are not visible in the GFP-PexRD31 in FigS6. If it is simply due to the magnification difference, then a higher magnification inset would be good to reassure the reader that all cells displayed punctae. If, instead, it was the case that not all cells did display punctae, then the authors should comment on that.

Comment 8.2: Furthermore, the authors examined all agroinfiltrations at 3 dpi, but expression of fluorescent protein fusions can generally be readily observed at 2 dpi, or even earlier. Were the punctae only visible at 3 dpi?

Authors response

Comment 8.1: This is indeed due to magnification differences. All the cells we imaged displayed puncta. A movie (Supplemental Movie 1) now accompanies the manuscript that shows a single plant cell at different magnifications and focal planes (the first image of that movie appears in Figure 6A). We hope this movie will help readers better appreciate the distribution, the mobility, and the intensity of the puncta in living cells. See Supplemental Movie 1, and see lines 378-380, 392-392. See also our response to Reviewer 3 comment 2.

Comment 8.2: Puncta were always visible, whenever we performed confocal microscopy assays (from 2 to 5 days-post infiltration). For practical reasons, we performed confocal assays three days after agroinfiltration. This notably allows us to accommodate week-end gaps within the experimental designs (i.e. infiltrate on Friday, image on Monday) and allows us to be consistent with confocal assays that include *Phytophthora infestans* infections (which usually requires 3 days to colonize *N. benthamiana* leaves to allow confocal microscopy assays).

Reviewer 1 comment 9 (minor point): Text and Fig S7B peroxisomes not peroxysomes.

Authors response: This has been corrected (see lines 399 and 738).

Reviewer 1 comment 10 (minor point)

Comment 10.1: Fig8: It would be good if panel B was larger. The top GFP panel in noticeably brighter than the others. Presumably the images were all collected with the same settings so the authors should note whether this higher brightness was a general property of co- expression with RD31 (in addition to the higher numbers of endosomes), suggesting stabilisation of the FYVE-tagged GFP.

Comment 10.2: If so it could be interesting to check if there were any FYVE domain proteins in the interactor list for RD31?

Authors response

Comment 10.1: We thank the reviewer for the suggestions for improvements. We have rearranged the figure 8 and made panel B (now panel A) larger. Images were indeed collected using the same settings, so the brightness of this GFP panel may reflect an increased abundance of the protein.

Comment 10.2: We have searched FYVE domain within the interactor protein sequences for RD31 using FYVE hmm from Pfam and HMMER software, but we did not find any.

<u>Reviewer #2:</u>

Reviewer 2 comment 1

Comment 1.1: My first point is mostly stylistic, but I think the authors would benefit from considering how they are presenting their large-scale datasets in Figure 2 and the associated Supplemental Figure S3. Right now these figures are incredibly densely packed, to the point it is difficult to easily see what they are attempting to demonstrate. My suggestion would be to a) use some other color than light gray to indicate the edges of these interaction maps as they are quite difficult to see in these figures.

Comment 1.2: Most of the discussion of the data in these figures involves interactions with small molecule transport, vesicle mediated transport, and cytoplasm-to-nuclear transport. Could the authors somehow highlight these by using different colors for the edges and/or GO term groups?

Comment 1.3: There is also some variation of the GO term groups based on the number of interacting host proteins that are present, but there is no elaboration of this (just varying numbers of points in these groups, to the extent that the number of proteasome- mediated ubiquitin-dependent components are virtually illegible). To me this should be a bit more clearly explained and presented.

Comment 1.4: Finally, some of the discussion of these results is a bit arbitrary because of the variable numbers of proteins in the different GO term groups. For example, on page 9 the authors focus on the large number of interactions observed between the PexRD12/31 effectors, but do not really mention the large number of AVR2 effectors as well. I also wonder about using the actual number of proteins in each of these GO groups vs the number of interactions observed. It is one thing to say that 7 of 9 protein import to nucleus components associate with the AVRvnt1 family, but this comparison gets more complicated when you are saying 24 out of 32 components are associated with the PexRD12/31 family, especially when also significant numbers of these interactions occur with the AVR2 and AVRblb2 families?

Authors response

Comment 1.1: We thank the reviewer for the suggestions on stylistic improvement for Figure 2. We consider it carefully and decided to keep the original rendering colors to reduce the complexity of the figure. However, we have deposited the network at The Network Data Exchange (NDEx) repository, and the interactive version of this effector-plant protein network depicted in Figure 2 is publicly available at repository with unique ID d60890f4- 5c95-11eb-9e72-0ac135e8bacf (http://public.ndexbio.org/viewer/networks/d60890f4- 5c95-11eb-9e72-0ac135e8bacf). Readers can download the original Cytoscape data file, render and analyze the figure to their specifications (see lines 929-932). Current figure could also be zoomed in to see the rendering of the edges in the figure as it is provided in high resolution.

Comment 1.2: Thank you again for your suggestion. A subnetwork of the targeted proteins in vesicle-mediated

process and effector families is depicted in Supplementary Figure 3 F and details are now given in Supplemental Data Set 3.

Comment 1.3: We now provide full GO terms for proteins and biological processes identified in the interactor screen (see Supplemental Data Set 2B). This data set, in combination with the interactive network, enables readers to analyze the data in detail.

Comment 1.4: The number of plant proteins in each GO biological process we included in our analyses were not arbitrary. We report only those identified in our screen, not all the proteins that are known to be involved in a particular process. Each effector family may target different biological processes, and a single biological process may be targeted by two or more effector families. We decided to focus our discussions on the interactors identified for PexRD12/31 family of effectors for novelty-related reasons. Indeed, Avr2 and Avrblb2 families have been already investigated in the past (see Saunders *et al.*, 2012 and Bozkurt *et al.*, 2011 for instance), while the investigative effort of the PexRD12/31 family was original.

Reviewer 2 comment 2

Comment 2.1: I am curious why the authors consider A1 and A2 subgroups to be a single group, and not two distinct groups? Based on the bootstrapping values it looks like these are significantly diverged from one another.

Comment 2.2: Also, it is important to provide all the bootstrapping values for the various branches of this phylogenetic tree.

Authors response

Comment 2.1: Thank you for your observation. Firstly, effectors in A1 subgroup have identical amino acid sequences in their effector domains which we used to construct the phylogenetic relationship, and so have those in A2. Essentially, A1 and A2 differ by just four amino acids. Secondly, A1 and A2 show more similarities to each other than those from other groups and are diverged from others on a single clade. Hence, we decided to group together in A as subgroups A1 and A2.

Comment 2.2: We have now provided all bootstrap values in the figure as well as a machine-readable tree file in Newick format (see Supplemental File 1).

Reviewer 2 comment 3: I think earlier in the description of the coIP/LC-MS/MS screen the authors indicated that they only considered interactors if the peptides ID'd were 80% identical and covered 80% of the protein sequence. I was therefore a bit confused when on page 13 they indicate that they only saw two peptides that matched the VAMP72x sequence. This should be clarified so that how these coIP interactions are being identified.

Authors response: We have now clarified this better in the text (see lines 188-192). We first filtered the protein hits with having at least two peptide hits with 90% probability and ion score of 40, and clustered the proteins based on 80% identity and 80% coverage.

Reviewer 2 comment 4

Comment 4.1: In Figure 4 I have two points. First, how is it that an anti-GFP VAMP72x is being co- IP'd when there is no significant IP of the anti-FLAG flag-tagged PITG_23074 protein? The FLAG tagged effector protein appears to be expressed quite poorly (third from bottom panel), and cannot be detected in the anto-FLAG IP (top panel), but this is one of the highest co-IPs in the anti-GFP (second from top panel)?

Comment 4.2: Second, to my eyes, the GFP-VAMP72x proteins appear to accumulate as a doublet between 40-55 kDa (second from bottom panel, and also to some degree third from top panel), but interestingly only a singlet is being co-IP'd with the FLAG-tagged effectors (second from top panel). This is not just a gel artifact, since the other band in the anti- GFP input (second from bottom panel) show no evidence of doublets (nor do the other background bands in the PITG_16427 lane of the anti-GFP IP [third from top panel). I wonder if the authors might want to look at this in a bit more detail. For sure the issue of the co-IP of VAMP72x with a non-existent FLAG-tagged effector should be addressed.

Authors response

Comment 4.1: It is possible that FLAG-PITG_23074 effector in question may not have been stable or transferred well to the blotting membrane during that particular experiment after being eluted from the affinity matrix. We have now included an immunoblot showing that FLAG-PITG_23074 (PexRD31) is detectable in a different experiment (see Supplemental Figure 5C). We observed this band three times (*i.e.* in three independent experiments). See also our response to Editor comment 5.

Comment 4.2: It is true in majority (two out of three) of the experiments that GFP-VAMP72x proteins were detected as doublet. It likely results from a partial degradation of the fusion protein during protein isolation. Note that when the protein was co-immunoprecipitated with effectors using anti-FLAG antibodies, only one band signal was detected indicating that other degradation product does not bind to the effector.

Reviewer 2 comment 5

Comment 5.1: In Supplemental Figure S6 the overlay with the brightfield actually obscures some of the information in these images. I would remove it.

Comment 5.2: In general, the images for Figures S6, Figure 6, Figure S7, and Figure 7 need to be improved. In Figure S6, the subcellular compartments imaged for the fluorescently- tagged PexRD31 appear to be qualitatively different from panel to panel. The sizes and distribution of these compartments varies dramatically. This is particularly apparent in figure S7 where the subcellular compartments in panels E, H, and I appear to be larger and much more uniform than in panels A, B, and D, where the structures are much more compact and more variable in size. There also seems to be quite a bit of background signal in the mCherry-PexRD31 panels in particular in these figures.

Comment 5.3: Finally, I'd like to address the comparison of "yellow" signal versus "red" signal in figures 6 and 7. I think the authors have simply tried to present the fluorescence in these panels based on the "color" of the FP they've attached to their marker. However, first, in a single channel image there is no utility to do this (the best is to present the image as greyscale, since that is what the camera is picking up, and also that will provide the best contrast in the image). But more importantly, second, colocalization studies have traditionally been presented in RGB format, where one channel is presented in red, a second in green, and a third in blue in an overlay. In this scenario, colocalization between a red protein and a green protein would present as yellow. But by overlaying a “yellow" panel with a "red" panel, the "yellow" signal is artificially presented as an overlay signal. This inappropriately indicates that the "yellow" protein colocalizes with the red protein, which is actually not the case in these figures. I agree there is likely some colocalization in these images, but it is not appropriate to combine a yellow and red panel. Better would be "green" and "red", or better yet, cyan and magenta as is instructed by the journal.

Authors response

Comment 5.1: We agree bright fields perturb overlay image interpretation. However, we would prefer to keep the overlay image with the bright field, as it actually informs readers about the health of the cells. More often than not, stressed *N. benthamiana* pavement cells can be identified by bright-field microscopy. Note that in all figures, the images showing only the GFP of mCherry signals allow readers to appreciate the distribution of the fluorescent signal without interference form the bright field. This being said, we have remade Supplemental Figures 6 and 7 (notably to address Editor comment 2) and, in the process, slightly reduced the brightness of bright field images.

Comment 5.2: We have prepared new figures that we hope are of better quality and correspond to a more uniform magnification. Note though that the variety of distribution patterns visible in Supplemental Figure 7 in the original manuscript originated from the very nature of the subcellular compartment we imaged (e.g., a *N. benthamiana* cell contains only one or two dozen of EXPO or peroxisomes, but hundreds of Golgi bodies or mitochondria). We also had to adapt the magnification to the size and the mobility of those compartments. We have revised the legend to clarify that specific point (see Supplemental Figure 7 legend).

Comment 5.3: We thank the reviewer for the suggestions on the use of colors in our figures. We have addressed the concerns appropriately (See our response to Editor comment 2).

Reviewer 2 comment 6:

Comment 6.1: I also want to point out some potential discrepancies in the identification of subcellular compartments associated with some of the markers used in this study. On page 17, the authors indicate that ARA6 and ARA7 label early and late endosomes and MVBs. I think the general consensus is that ARA6 and ARA7 label endosomes, and particularly do not label "early endosomes" which at this point are considered TGN/EE, which are distinct compartments. It is probably better to refer to these compartments as "endosomes and MVBs.

Comment 6.2: Also, I would urge significant caution in identifying RabC1 labeling as a "post- Golgi/endosomal network" compartment. This designation is an artifact from early classification of plant Rab GTPases based on localizations assigned to yeast and mammalian localizations. RabC GTPases, which are within the same phylogenetic clade as Rab18 GTPases from mammals, were classified as post-Golgi/endosomal based on early reports in polarized mammalian epithelial cells. These reports in the early 90s were likely incorrect, and there is good evidence that Rab18 now localizes to ER-derived lipid bodies and possibly peroxisomes (also ER-derived). What the subcellular compartment is that RabC GTPases localizes to in plants is unclear, but I would urge the authors to refrain from calling these post-Golgi/endosomal compartments because there is simply little evidence to support this in the plant literature at this point.

Authors response

Comment 6.1: We agree with the reviewer. We now refer to compartments labelled by ARA6 and ARA7 as 'endosomes' in the manuscript.

Comment 6.2: Again, we agree with the reviewer. We no longer refer to RabC1-positive vesicles as components of the post-Golgi network in the text, but simply refer to RabC1-positive bodies. (see also our response to Editor comment 3).

Reviewer 2 comment 7: In Figure 7 it would be helpful to present lower magnification images so that these haustorial structures can be placed in context of the cell. Right now these are cropped so tightly it is difficult to see the actual structures, not to mention place them in context of the plasma membrane, and other potential structures in their vicinity.

Authors response: These points have now been addressed (see our detailed responses to Editor comment 2 and Editor comment 4). We have uncropped the images presented in the revised manuscript, and included when possible low magnification images to show the broader cellular context (see Figure 7A for instance).

Reviewer 2 comment 8

Comment 8.1: In Figure 8 also, the images are too small to allow judgement of the increased FYVE- labeled compartments.

Comment 8.2: I also am curious whether 3D z-stacks are being used to quantify total FYVE-labeled compartments in these cells? If not, the authors will need to ensure that they are presenting z-planes that are consistent from image to image (which is likely to be technically difficult). If they are not doing this, how are they sure these increased numbers of compartments they are counting are representative of the actual cellular number? Related to this, do the authors also see increased numbers of ARA6/ARA7- labeled compartments? These Rab GTPases specify PI3P labeled compartments, they should see a concomitant increase in ARA6/7 compartments if these endosomal compartments are really increasing in number.

Authors response

Comment 8.1: We thank the reviewer for this suggestion. We have rearranged the figure accordingly and made panel B (now panel A) larger (see also our response to Reviewer 1 Comment 10).

Comment 8.2: Indeed, 3D z-stacks were used to quantify total FYVE-labeled compartments. Images shown in Figure 8 panel A are all maximum intensity projections and now include total z-stack volume. Figure legend has been amended to make it clear to readers that z- stacks were used. Studying this effectors' impact on ARA6/ARA7-labeled compartments is a good suggestion, which will be taken into consideration for future investigations.

Reviewer 2 comment 9

Comment 9.1: In Figure 9, I'm not sure I agree with the statement that increased numbers of PI3P- labeled compartments is what they are observing. To me it looks like they are seeing more "obvious" labeling of PI3P compartments, which might actually be due to aggregation of PI3P-labeled compartments.

Comment 9.2: I don't think there is much connection between the low magnification data and the high magnification images presented in Figure 9B. The authors should attempt to refer from low-mag images in 9A to directly link these to high-mag images in 9B.

Authors response

Comment 9.1: We agree with the reviewer. We indeed think that *P. infestans* infection triggers a clustering of PI3P compartments, making them appear as large bright puncta (as documented in Figure 9). We have modified the text to use "aggregate" to describe the puncta observed in Figure 9. See lines 490 & 1481.

Comment 9.2: We respectfully disagree on this point. The puncta visible in Figure 9B are similar to those in Figure 9A (that was obvious during experimentations under the microscope, when puncta were easy to track while changing objectives). To allow readers to better appreciate the mobility and size of the puncta shown in Figure 9B, we have included a movie in the revised manuscript (the first image of the movie being the image shown in Figure 9B). See Supplemental Movie 3 and see line 507.

Reviewer 2 comment 10: I think the discussion is fine, but I would again urge the authors to be more circumspect in their description of subcellular membrane markers in the first paragraph of page 28. This work can provide a really nice platform to bridge the plant immunity and cell biology fields in plants, but that will rely on accurately assigning subcellular compartments.

Authors response: We agreed with the reviewer and we have addressed this concern (see also our response to Reviewer 2 comment 6 and Editor comment 3). We have modified the discussion accordingly in the revised manuscript.

Reviewer #3:

Reviewer 3 comment 1: Quantitative data for colocalization are missing

Authors response: Thank you for this reminder. We have included quantitative data in the revised manuscript (see our detailed response to Editor comment 1).

Reviewer 3 comment 2: Images for PexRD31 presented throughout the paper do not show a consistent pattern

Authors response: We now have included a movie showing PexRD31 mobile bodies (Supplemental Movie 1) and detailed explanation in our response to Reviewer 1 comment 8. In brief, PexRD31 shows consistent pattern in cells; but low magnification and central focal plane images are not sufficient for visualizing PexRD31 mobile bodies. Supplemental Movie 1 clarifies this point.

Reviewer 3 comment 3: How much of PexRD31 is at the PM and how much is associated with vesicles?

Authors response: This is an interesting question, for which we do not have a clear answer (neither an accurate way to answer it). Indeed, the size and nature of the cell compartments where PexRD31 accumulates makes it difficult to quantify its distribution, either by microscopy or cell fractionation. This being said, our confocal microscopy data seem to show a stronger fluorescent signal at the plasma membrane compared with the RabC1-positive bodies (see Supplemental Movie 1, for instance). For the sake of peer discussion, we would thus propose that PexRD31 primarily accumulates at the plasma membrane.

Reviewer 3 comment 4: Using RabC1 as the sole marker for the post-Golgi endosomal system is surprising as it has been shown to behave very similar to other TGN/EE-markers including SYP61, VHA-a1 and RabA2/3.

Authors response: This point has also been brought up by another reviewer and we have now addressed it carefully (see our responses to Reviewer 2 comment 6 and Editor comment 3). In short, we have revised our statements regarding the nature of RabC1-positive bodies and modified our conclusions, which reduce our emphasis on TGN and remove the need to use additional TGN makers.

Reviewer 3 comment 5: What is known about the localization of these markers in N. benthamiana?

> **Authors response: To the best of our knowledge, all three markers label TGN/EE in agroinfiltration & confocal microscopy assays in *N. benthamiana*. They are notably used to delineate the endocytic routes of immune receptor kinase upon ligand recognition (Mbengue et al., 2016. PNAS 113:11034–11039).**

TPC2020-LSB-00804R1 2nd Editorial decision – *acceptance pending* Feb. 8, 2021

We are pleased to inform you that your paper entitled "Host-interactor screens of *Phytophthora infestans* RXLR proteins reveal vesicle trafficking as a major effector-targeted process" has been accepted for publication in The Plant Cell, pending a final minor editorial review by journal staff.

Final acceptance from Science Editor Feb. 19, 2021
